# Advances in mercury ion sensing using BODIPY-based compounds: a sexennial update

**DOI:** 10.1039/d5ra01232e

**Published:** 2025-04-01

**Authors:** Supriya Routray, Subhadeep Acharya, Laxmipriya Nayak, Simran Pattnaik, Rashmirekha Satapathy

**Affiliations:** a Department of Chemistry, Ravenshaw University Cuttack-753003 Odisha India rashmi@ravenshawuniversity.ac.in

## Abstract

Pollution from mercury ions (Hg^2+^) continues to pose a significant threat to the environment and public health because of its extreme toxicity and bioaccumulative nature. BODIPY-based compounds are emerging as strong candidates for creating selective and sensitive chemosensors for mercury ion detection. Their structural tunability facilitates the introduction of various functional groups, improving their binding affinity and specificity toward mercury ions. This review elucidates various sensing mechanisms and provides comprehensive insights into the performance of these sensors, particularly with regard to selectivity, sensitivity, and detection limits. The synthetic routes for synthesizing the chemosensors are mentioned in detail. Given their reliability and flexibility, BODIPY-based sensors are poised to make significant contributions in the fields of both sensors and analytical chemistry.

## Abstract 2

This review presents recent advancements (2019–2024) in BODIPY-based chemosensors for selective detection of mercury ions.

## Graphical abstract

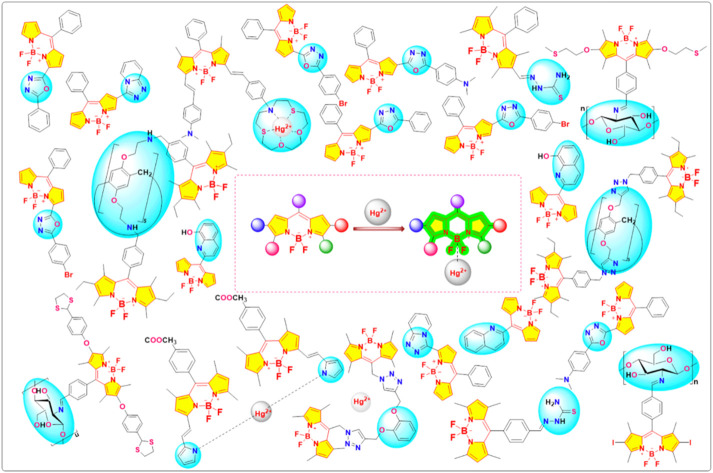

## 1. Introduction

**Fig. 1:**
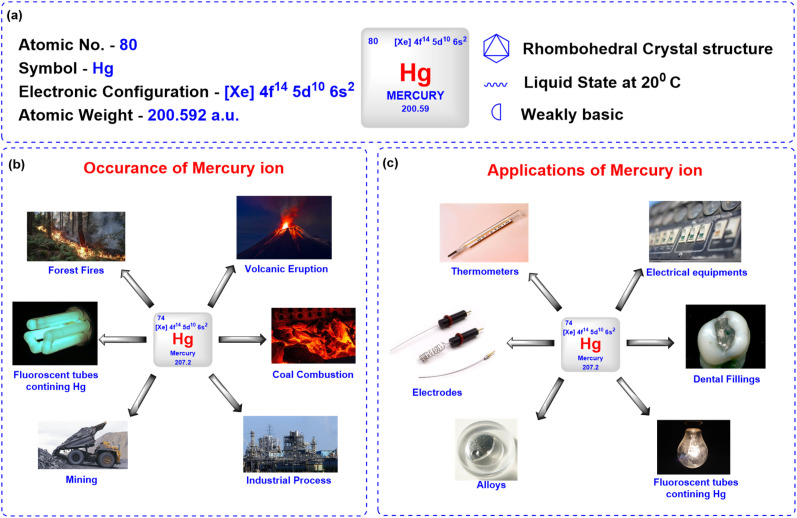
(a) Overview of mercury metal; (b) occurrence of mercury; (c) applications of mercury.

Mercury ions pose a significant threat to environmental safety and human health due to their toxicity and tendency to bioaccumulate in both natural and artificial environments.^1^ Naturally occurring in the Earth's crust, mercury exists in various forms such as elemental form (liquid metal), inorganic compound form (*e.g.*, mercuric chloride, mercuric sulfide), and organic compound form like methylmercury, which is a potent neurotoxin that accumulates in food chains. Additionally, mercury can appear as ions, mainly mercurous (Hg_2_^2+^) and mercuric (Hg^2+^).^2^ Natural sources, such as volcanic eruptions and wildfires, contribute to mercury emissions; however, human activities, including mining, coal combustion, industrial processes, and waste incineration, are the primary contributors to mercury pollution (Fig. 1).^3^

Mercury has been widely utilized in various applications due to its distinct chemical and physical properties, including its liquid state at room temperature and high density. In laboratories, mercury is commonly used in the manufacturing of thermometers, barometers, and diffusion pumps, as well as in sphygmomanometers for medical purposes (Fig. 1).^3^ Its excellent electrical conductivity makes it valuable in the production of electrical switches, relays, and other electronic devices. Mercury serves as a liquid electrode in electrochemical processes, such as the industrial synthesis of chlorine and sodium hydroxide.^4^ In the chemical industry, mercury acts as a catalyst in various reactions, while its ability to form amalgams with metals like gold and silver has historically been exploited in dental fillings and gold extraction processes. Additionally, mercurous chloride, a mercury compound, has been used in medicine as a purgative and as a reference electrode (calomel) in electrochemical measurements. However, due to its toxicity, the use of mercury in many of these applications is now regulated or being phased out in favour of safer alternatives.

Despite its utility in various sectors, mercury's toxic effects are profound. The World Health Organization (WHO) and the US Environmental Protection Agency (EPA) set strict allowable limits for mercury in drinking water, highlighting the serious risks associated with exposure. The World Health Organization (WHO) has established a permissible limit of mercury ions in drinking water at 0.5 μg L^−1^. In contrast, the US Environmental Protection Agency (EPA) has determined the acceptable limit for inorganic mercury in drinking water to be 0.002 mg L^−1^ (2 ppb).

**Fig. 2:**
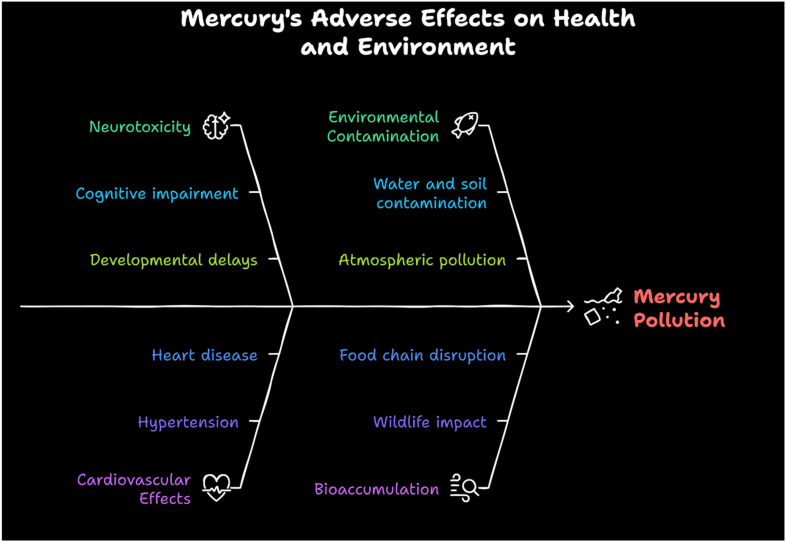
Adverse effects of mercury on health and environment.

Exposure to mercury has profound and far-reaching consequences on human health and the environment. Mercury can severely damage the central nervous system, kidneys, and immune system, leading to cognitive impairments, neuromuscular disorders, and kidney dysfunction.^5^ Methylmercury, a highly toxic organic form, is particularly harmful to developing fetuses and infants, as it can cross the placental barrier and disrupt normal brain development, causing long-term neurological damage.^6–9^ Acute mercury exposure can result in symptoms such as headaches, tremors, insomnia, and respiratory irritation, while chronic exposure may lead to Minamata disease, characterized by severe motor and sensory impairments (Fig. 2).^10–13^ Mercury also disrupts proteins and enzymes, causing oxidative stress, endocrine disturbances, and altered gut flora.^14^ Additionally, it acts as an epigenetic toxicant, potentially influencing gene expression in future generations. Environmental contamination from mercury persists throughout the food chain, with bioaccumulation in fish posing a significant risk to human populations that rely on seafood as a dietary staple.^15,16^ The severity of these adverse effects underscores the need for stringent regulatory measures and innovative detection methods to mitigate mercury contamination.

**Fig. 3:**
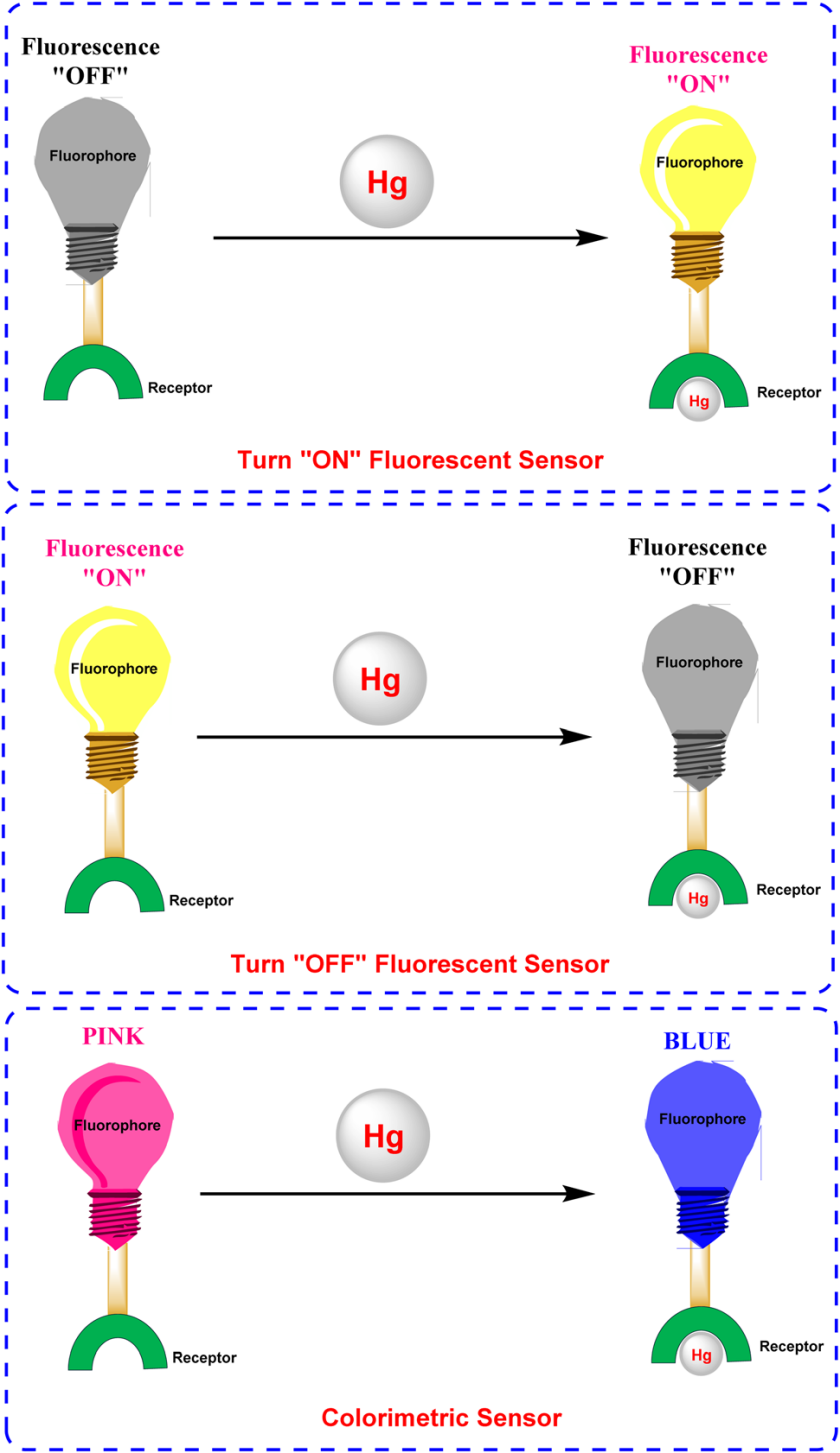
Schematic overview of various types of colorimetric and/or fluorogenic receptors for the detection of mercury ions; (top) turn “ON” fluorescent sensor; (middle) turn “OFF” fluorescent sensor; (bottom) colorimetric sensor.

Fluorescent sensors have emerged as a compelling alternative^17^ to conventional methods such as reduced graphene oxide field-effect transistor (rGO FET), cold vapor integrated quartz crystal microbalance (CV-QCM), electrochemical analysis, atomic absorption spectrometry, gas chromatography-triple quadrupole mass spectrometry (GC-MS/MS), mercury analyzers, and mass spectrometry, which often require expensive equipment and are too cumbersome for on-site or live-cell detection.^18–24^ These sensors are particularly advantageous for metal ion detection because of their simplicity, quick response, and low detection limits. Unlike traditional approaches, fluorescent sensors enable real-time monitoring without complex sample preparation, making them ideal for dynamic imaging in living cells and a wide range of biomedical and environmental applications.^25–31^ Several mechanisms facilitate analyte detection, including ET (energy transfer), PET (photo-induced electron transfer),^32–34^ CHEF (chelation-enhanced fluorescence),^35–37^ FRET (Förster resonance energy transfer)^38–41^ and ESIPT (excited state intramolecular proton transfer).^42,43^ By leveraging carefully designed fluorophore–ligand systems, these sensors achieve extreme sensitivity and outstanding selectivity, reducing interference from different metal ions and ensuring accurate detection of low mercury concentrations in aqueous environments (Fig. 3). Furthermore, their ability to integrate into portable devices or test strips enhances their practicality and cost-effectiveness for on-site metal analysis, underscoring their versatility in real-world scenarios.^44,45^ A notable challenge in the realm of metal ion sensing is the targeted identification of mercury ions. Consequently, the development of differential sensing methodologies for Hg^2+^ has emerged as a significant area of research. To this end, diverse rational design strategies utilizing fluorophore scaffolds such as cyanine, rhodamine, fluorescein, coumarin, anthracene, and BODIPY-based derivatives have been instrumental in creating novel functional fluorescent probes capable of sensitively and selectively detecting mercury (Hg^2+^) ions.^46–51^

**Fig. 4:**
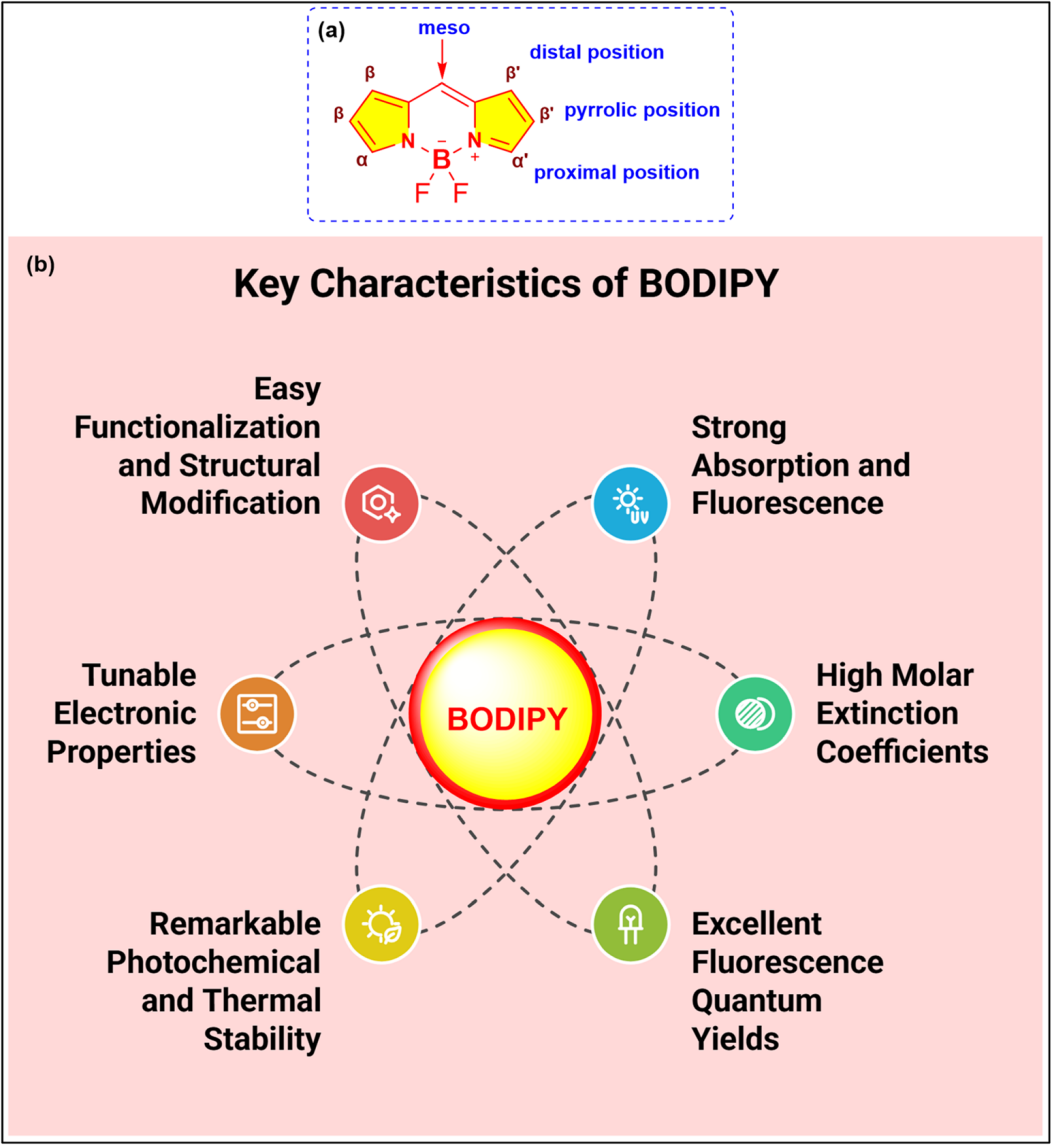
(a) Structure and different substitution positions of BODIPY; (b) key characteristics of BODIPY.

**Fig. 5:**
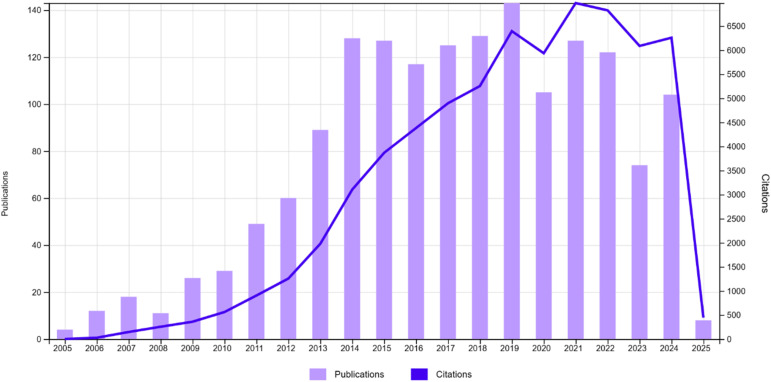
Analysis of publications and citations of BODIPY-based fluorescent sensors from 2015–2025 (Web of Science).

4,4-Difluoro-4-bora-3*a*,4*a*-diaza-*s*-indacene (BODIPY) is a highly versatile fluorophore renowned for its exceptional photonic and electronic characteristics (Fig. 4).^52,53^ Since its discovery in 1968 by Treibs and Kreuzer,^54^ BODIPY-based compounds have garnered extensive attention across diverse fields, including biochemistry, materials science, physics, and electronics.^55^ Its unique features, such as excellent absorption and emission in the UV-vis region, high fluorescence quantum yields, and molar extinction coefficients with remarkable photochemical and thermal stability, have established BODIPY as a pivotal building block in the development of advanced fluorescent materials.^56,57^ Furthermore, its highly conjugated core and tunable electronic properties make it an ideal candidate for functionalization and structural modification.^58,59^ Moreover, anchoring BODIPY compounds onto a solid support offers several practical benefits. This approach not only enhances sensor performance by increasing operational stability but also minimizes issues such as aggregation and leaching while enabling more straightforward integration into portable devices and sensor platforms. An analysis of the literature on BODIPY-based fluorescent sensors showed that 1183 research papers were published over the past decade (2015–2025). The analysis reveals an upward trend in the number of publications and citations over time, indicating a substantial increase in interest in this rapidly growing field (Fig. 5).

The versatile characteristics of BODIPY have been harnessed to design BODIPY-based fluorescent sensors capable of detecting a broad spectrum of analytes, including anions,^60^ cations,^61,62^ phosgene,^63^ and nerve gases.^64,65^ BODIPY scaffolds stand out as particularly promising for mercury ion detection as these sensors exhibit fluorescence changes, manifested by turn-on or turn-off or colorimetric responses, providing a simple and visually observable detection mechanism. Incorporating specific receptors or functional groups into the BODIPY scaffold enables the creation of chemosensors that exhibit excellent selectivity and sensitivity towards mercury ions.

**Fig. 6:**
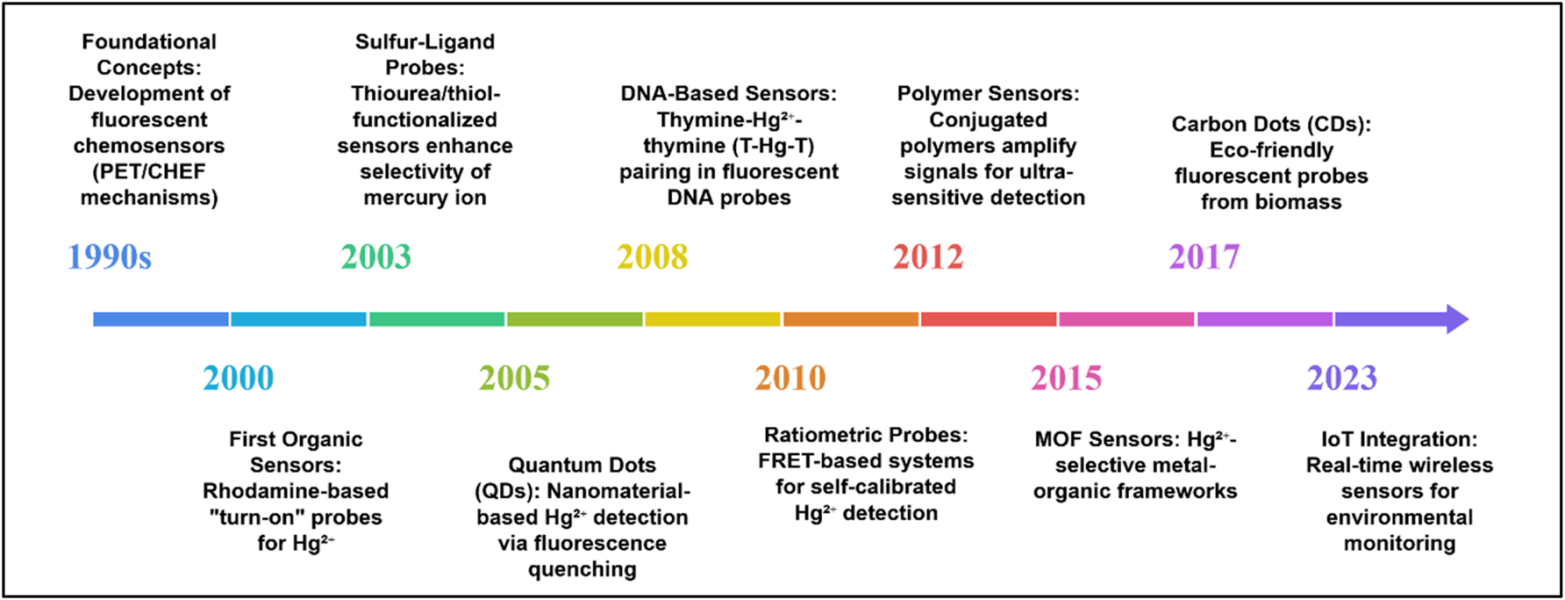
Roadmap for the evolution of fluorescent chemosensors for mercury ion detection.

While a number of comprehensive reviews in the last decade exist detailing the broader development of BODIPY-based sensors, a dedicated analysis focusing specifically on the synthesis and mechanisms of BODIPY-based mercury ion sensors remains absent from the literature.^56,66–69^ This review addresses this gap by examining recent advancements (2019–2024) in BODIPY-based chemosensors designed for mercury ion detection. To begin, we present and outline the general principles of designing effective fluorescent imaging probes, as well as their various sensing methods, to better understand rational design approaches and discuss how these fluorescent probes interact with analytes. Then, we comprehensively analyze their colorimetric and fluorogenic properties, utilizing data obtained from different *in situ* and *ex situ* spectroscopic techniques like fluorescence, UV-vis, and NMR spectroscopic techniques across diverse solvent systems. The central objective is to provide an in-depth understanding of these sensors, with a particular emphasis on synthetic strategies, binding mechanisms, complex stoichiometry, and the thermodynamics governing host–guest interactions. The review is structured thematically, with subsections dedicated to different types of substitution on the BODIPY core. Ultimately, we wrap up with a discussion of the realities and future challenges in this field (Fig. 6).

## 2. Photophysical sensing mechanism of fluorescent sensors

**Fig. 7:**
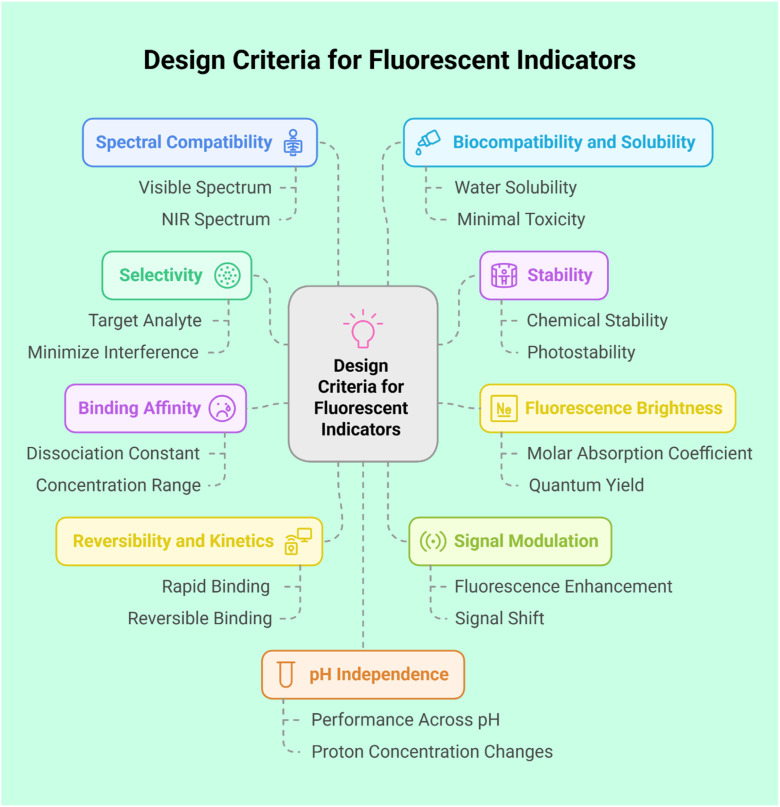
Design criteria for fluorescent sensors.

The design of fluorescent sensors prioritizes key attributes such as selectivity, stability, sensitivity, fluorescence brightness, signal modulation, pH independence, spectral compatibility, and binding affinity to ensure accurate and dependable analyte detection (Fig. 7).^70^ These indicators operate by exhibiting changes in fluorescence properties, such as intensity or emission wavelength, upon interaction with a target analyte. Central to their functionality are photophysical sensing mechanisms, which enable the conversion of chemical interactions into measurable optical signals, such as fluorescence or absorption. These mechanisms offer high sensitivity, selectivity, and the capability to monitor analytes in real time. Commonly utilized mechanisms include PET (photo-induced electron transfer), CHEF (chelation-enhanced fluorescence), FRET (Förster resonance energy transfer), ICT (intramolecular charge transfer), AIE (aggregation-induced emission), and ESIPT (excited state intramolecular proton transfer), each offering distinct advantages tailored to specific sensing applications.^71^ Below is a concise overview of various fluorescence-sensing mechanisms.

### 2.1 Photoinduced electron transfer (PET)

**Fig. 8:**
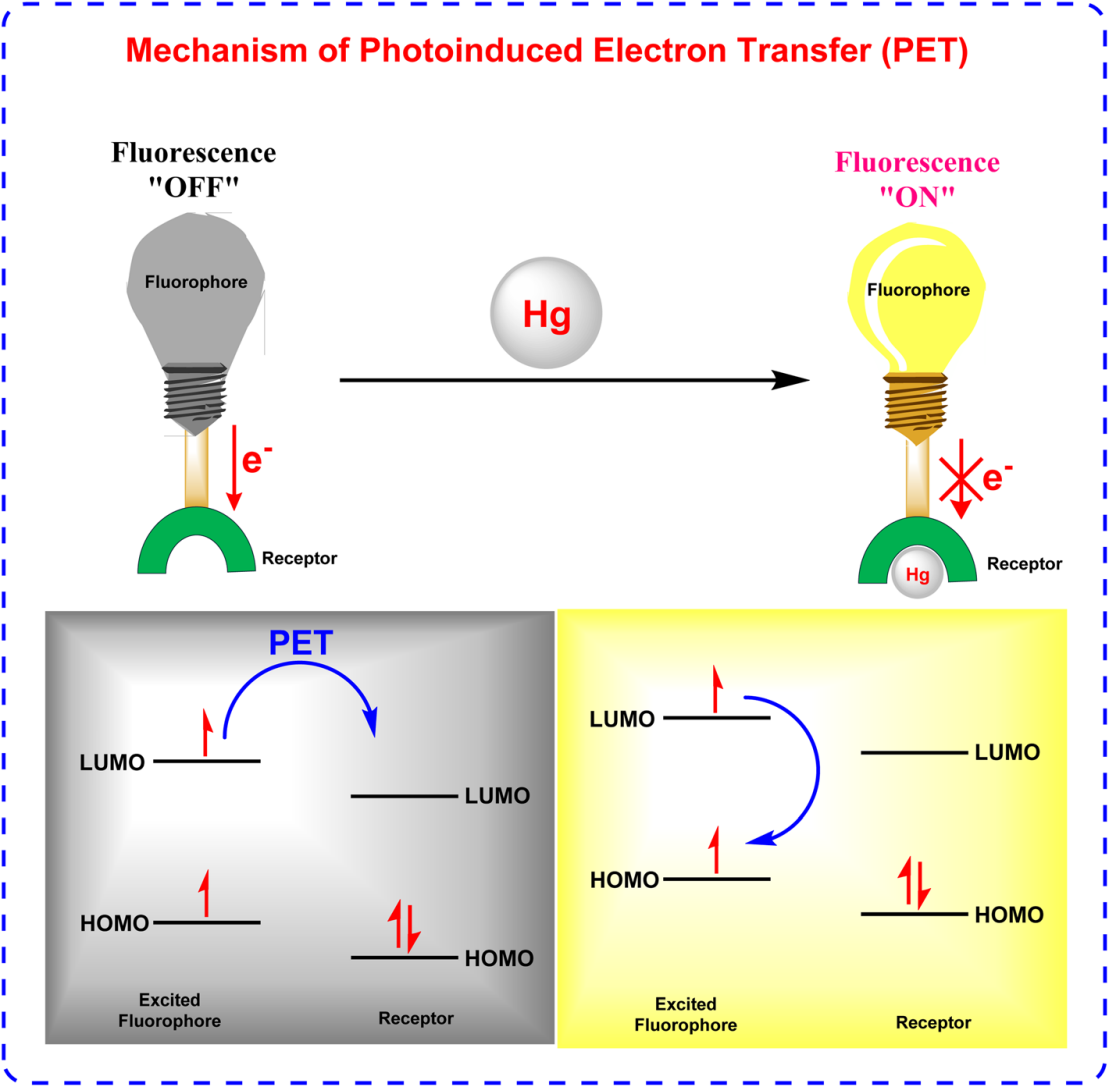
Mechanism of photoinduced electron transfer (PET).

Photoinduced Electron Transfer (PET) is a fundamental mechanism in fluorescence sensing, characterized by the transfer of an electron between a fluorophore and a recognition unit, which modulates the fluorescence signal. Typically, PET-based systems are designed with a fluorophore linked to a recognition site *via* a spacer, allowing for selective interactions with target analytes (Fig. 8). The process is governed by the alignment of molecular orbital energy levels, where the highest occupied molecular orbital (HOMO) of the recognition unit lies between the HOMO and lowest unoccupied molecular orbital (LUMO) of the fluorophore. In the absence of analyte binding, PET leads to fluorescence quenching through nonradiative electron transfer. However, upon analyte binding, the energy levels are altered, inhibiting PET and restoring fluorescence. This mechanism allows PET sensors to function as “on-off” or “off-on” switches, making them highly sensitive tools for detecting ions, biomolecules, and environmental changes.^72^

### 2.2 The intramolecular charge transfer (ICT)

**Fig. 9:**
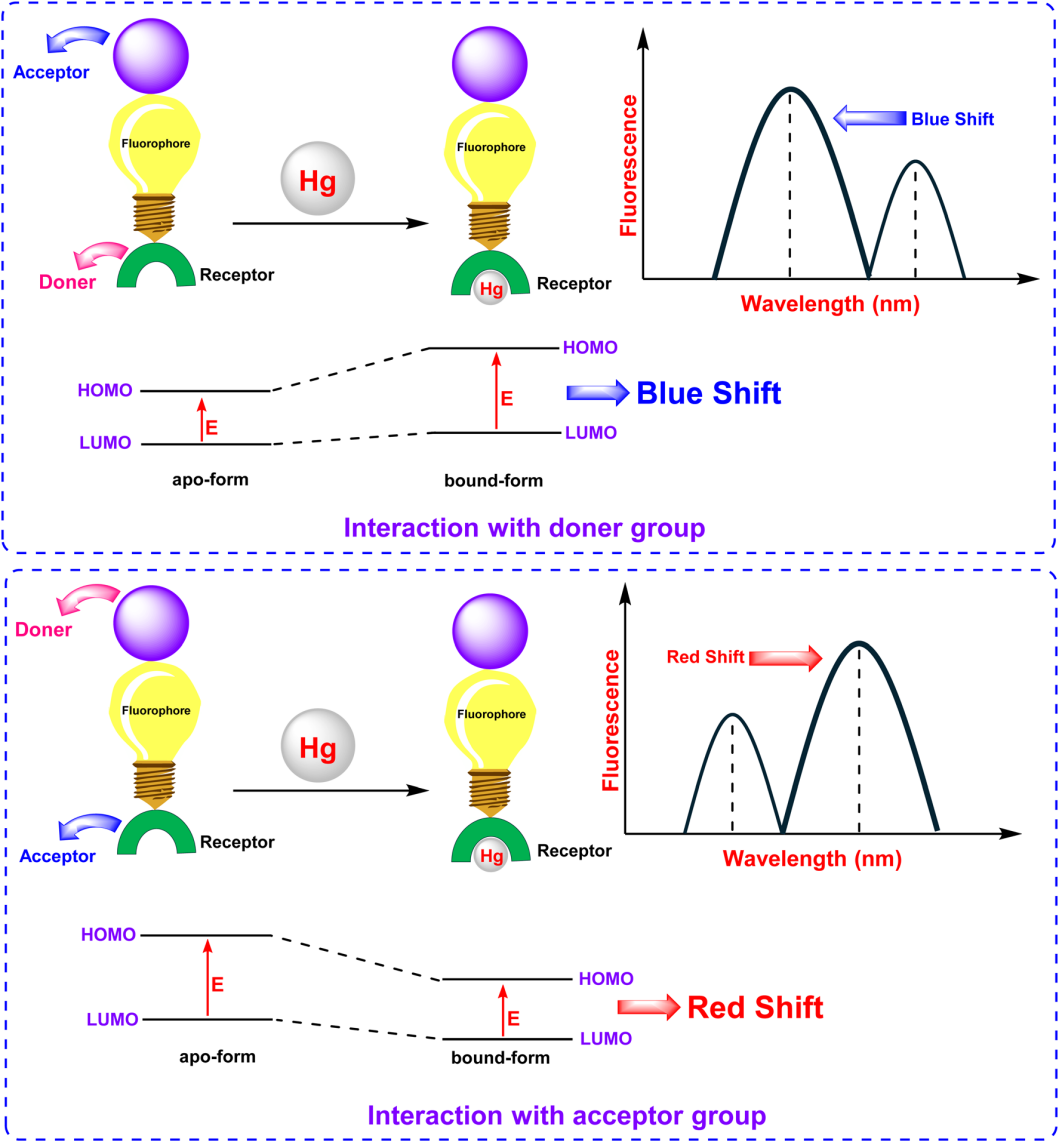
Mechanism of Intramolecular Charge Transfer (ICT).

The Intramolecular Charge Transfer (ICT) mechanism, first introduced by Valeur, serves as a foundational strategy for designing fluorescent chemosensors. This approach relies on a conjugated π-system that integrates an electron-donating unit (D) and an electron-accepting unit (A), thereby forming a “push–pull” electronic configuration. Upon excitation, charge redistribution occurs, with the HOMO localized near the donor and the LUMO near the acceptor, generating an intense dipole moment (Fig. 9).^73^ Analyte binding modulates this electronic system: interactions at the donor site reduce its electron-donating capacity, weakening the ICT and inducing a blue shift in absorption/emission spectra. Conversely, interactions at the acceptor site enhance ICT, producing a red shift. These spectral shifts, coupled with changes in fluorescence quantum yields and lifetimes, enable ratiometric sensing—a method resistant to environmental interference. The direct conjugation of the receptor and fluorophore (without spacers) ensures efficient electronic communication, allowing precise modulation of optical properties. ICT-based probes are widely employed for detecting cations, protons, and polar analytes, leveraging their sensitivity to solvent polarity and analyte-induced electronic perturbations.

### 2.3 Fluorescence resonance energy transfer (FRET)

**Fig. 10:**
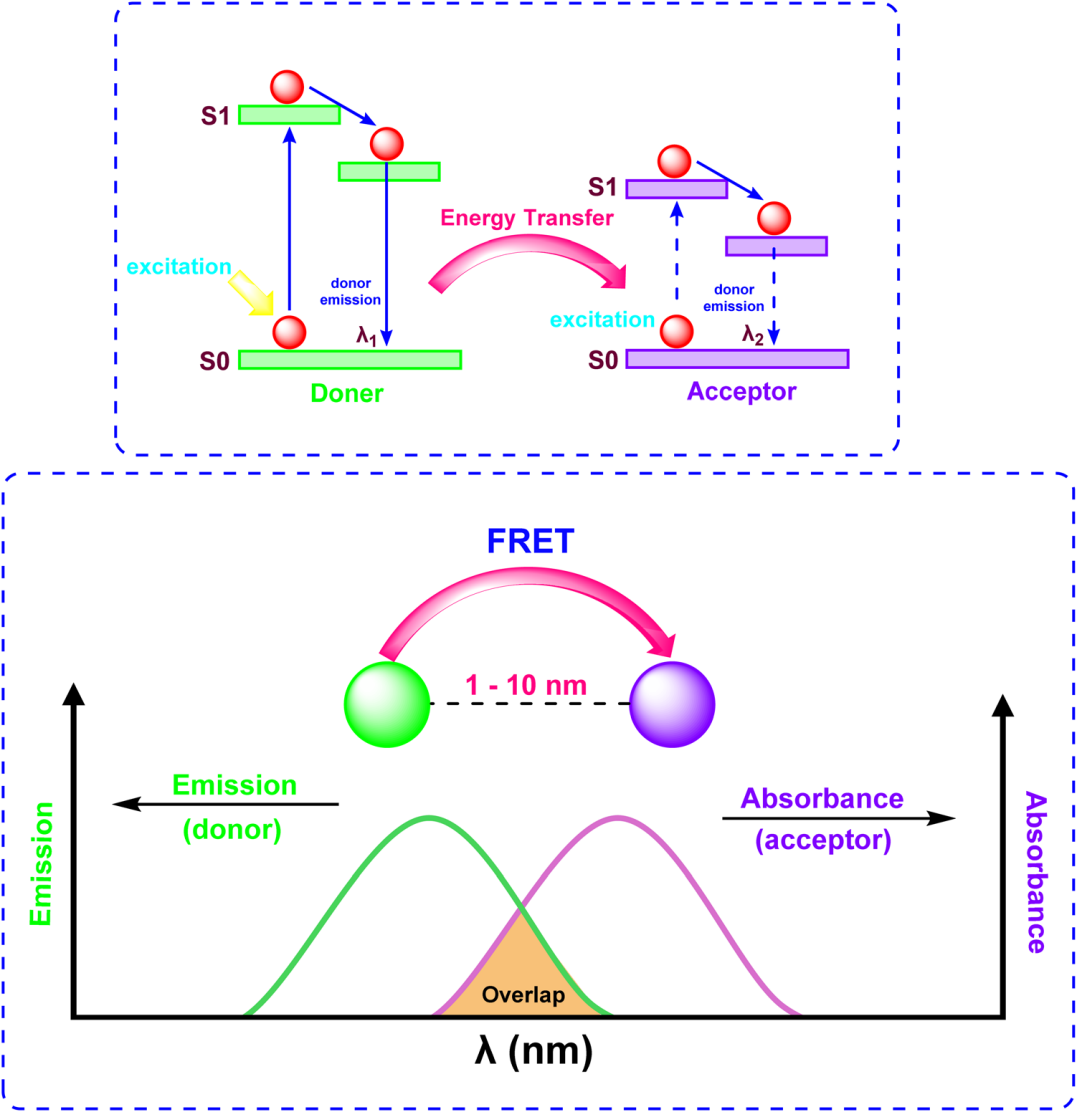
Mechanism of Fluorescence Resonance Energy Transfer (FRET).

Fluorescence Resonance Energy Transfer (FRET) is a photophysical phenomenon involving non-radiative energy transfer between two chromophores, an acceptor and a donor mediated by dipole–dipole coupling (Fig. 10).^38^ Energy transfer happens when the donor's emission spectrum coincides with the acceptor's absorption spectrum, allowing for transfer over distances of 1–10 nm. FRET efficiency (*E*) exhibits an inverse sixth-power dependence on the donor–acceptor separation distance (*E* ∝ *r*^−6^), rendering it highly sensitive to nanoscale molecular proximity and conformational changes. This distance-dependent mechanism facilitates real-time monitoring of biomolecular interactions, such as protein binding and nucleic acid dynamics, with applications spanning biological imaging and ratiometric sensing of analytes like Hg^2+^ and Zn^2+^. The absence of photon emission during energy transfer distinguishes FRET from conventional fluorescence methods, allowing precise spatial resolution in dynamic molecular systems.

### 2.4 Excited-state intramolecular proton transfer (ESIPT)

**Fig. 11:**
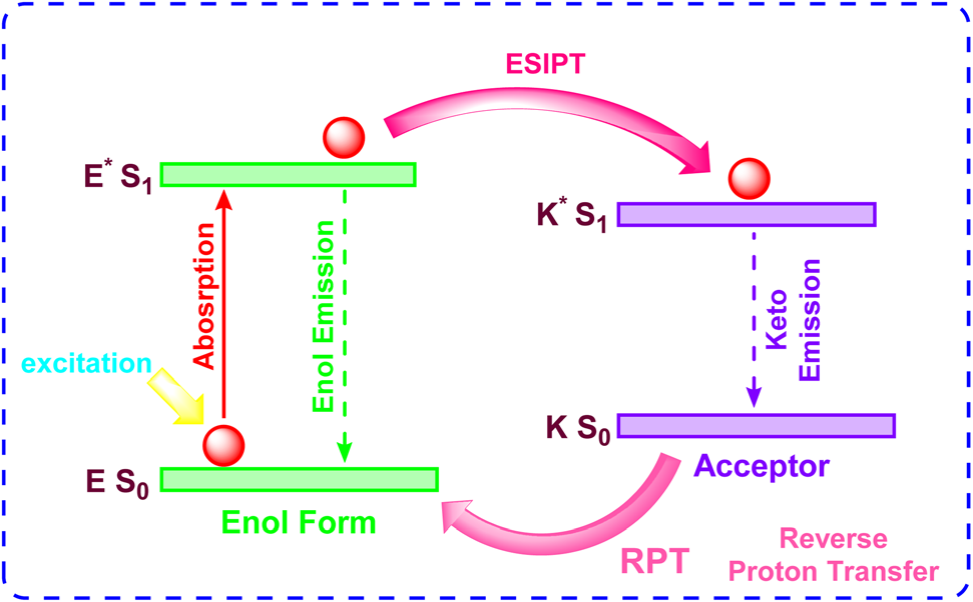
Mechanism of Excited-State Intramolecular Proton Transfer (ESIPT).

Excited-State Intramolecular Proton Transfer (ESIPT) is a photophysical process characterized by the ultrafast relocation of a proton between adjacent atoms within a molecule following photoexcitation, leading to tautomerization between enol (E) and keto (K) forms (Fig. 11).^74–76^ This mechanism is mediated by a pre-existing intramolecular hydrogen bond (H-bond) between a proton donor group, such as hydroxyl or amino, and an acceptor site, typically carbonyl oxygen or imine nitrogen. Upon excitation, the strengthened H-bond and charge redistribution in the excited state lower the energy barrier for proton transfer, enabling spontaneous conversion from the enol (E*) to keto (K*) tautomer in the S_1_ state. The process follows a four-level photocycle (E → E* → K* → K), where emission occurs from the keto form, resulting in significant Stokes shifts (>200 nm) due to significant structural and electronic differences between the tautomers. ESIPT-based systems exhibit enhanced photostability and reduced excited-state reactivity, making them ideal for designing fluorescent probes with environmental sensitivity. Derivatives like 2-(2-hydroxyphenyl)benzothiazole (HBT) demonstrate substituent-dependent dynamics, where electron-donating groups optimize H-bond strength and modulate energy barriers (∼2–3 kcal mol^−1^), as validated by time-dependent density functional theory (TDDFT) and experimental spectroscopic analyses. These properties enable applications in ratiometric sensing, bioimaging, and optoelectronic devices.

## 3. Recent advances in BODIPY-based mercury ion sensors

### 3.1 Pillararene-BODIPY-based mercury ion sensor

Pillararenes represent a class of macrocyclic compounds characterized by a framework composed of hydroquinone or dialkoxybenzene units, typically ranging in number from five to ten. These units are interconnected at the para positions *via* methylene bridges. The inherent synthetic accessibility of pillararenes facilitates the attachment of diverse functional groups to their periphery. This modification strategically provides tailored interaction sites for specific applications such as cation recognition.^77^

**Fig. 12:**
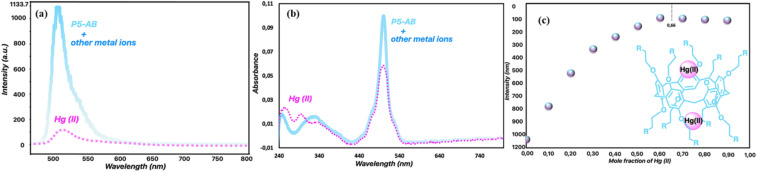
(a) Fluorescence spectra of probe 9 in H_2_O/CH_3_CN (1 : 1). (b) Absorption spectra of probe 9 in H_2_O/CH_3_CN (1 : 1). (c) The interaction between probe 9 and Hg^2+^ in H_2_O/CH_3_CN (1 : 1). Reproduced with permission from ref. 78. Copyright 2023, Elsevier.

**Scheme 1:**
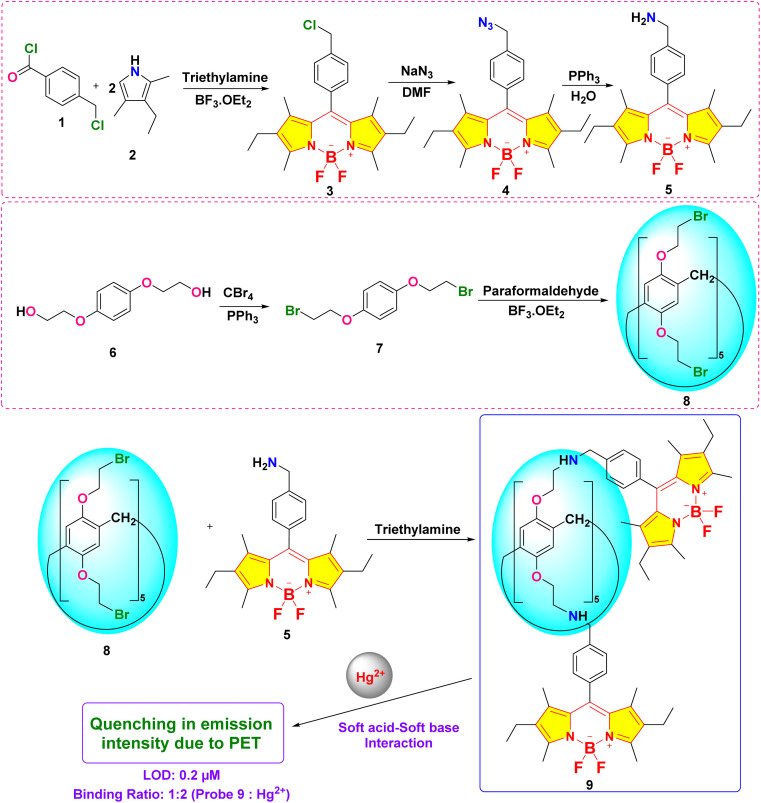
Synthetic route for probe 9.

In 2023, Yemisci and colleagues created a new macrocyclic fluorescent probe 9 by integrating pillar[5]arene with BODIPY components for the specific detection of Hg^2+^ in a 1 : 1 (v/v) acetonitrile and water solution.^78^ Spectroscopic investigations revealed a photoinduced electron transfer (PET) mechanism between the doner atoms of probe 9 and Hg^2+^ ions, supported by fluorescence quenching at 515 nm (1130 units to 110 units) and decrease in the UV-vis absorption spectra at 500 nm, which is attributed to complex formation between probe 9 and Hg^2+^ ion (Fig. 12). The sensor, through fluorometric analysis, demonstrated a detection limit of 0.2 μM, and Job plot analysis confirmed a 1 : 2 binding stoichiometry. It has been found that probe 9 captures the Hg^2+^ ion at two separate sites: the amino groups of BODIPY units and the oxygen atoms in the pillar[5]arene framework. The heightened sensitivity of probe 9 to Hg^2+^ is attributed to the soft acid–soft base interactions (Scheme 1).

**Scheme 2:**
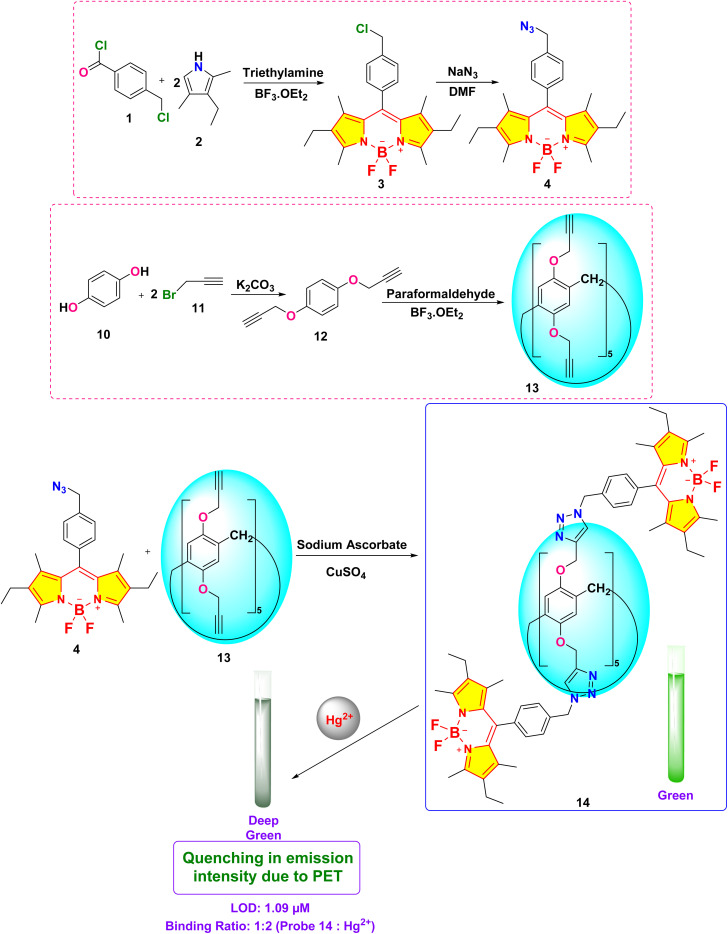
Synthetic route for probe 14.

**Fig. 13:**
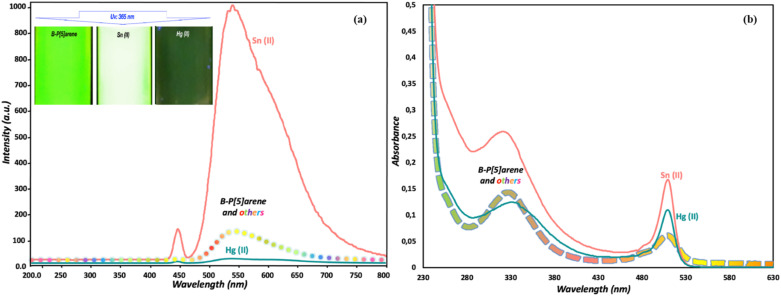
(a) Emission spectra of probe 14 in H_2_O/CH_3_CN (1 : 1) (1 μM) in presence of various metal ions. (b) The absorption spectra of probe 14 in H_2_O/CH_3_CN (1 : 1) (1 μM) in presence of various metal ions. Reproduced with permission from ref. 62. Copyright 2022, Elsevier.

Kursunlu and colleagues developed a novel dual-channel macrocyclic sensor (probe 14) by combining BODIPY fluorophores with pillar[5]arene for the selective detection of Hg^2+^ and Sn^2+^ in aqueous-acetonitrile media (1 : 1 v/v) (Scheme 2).^62^ In the fluorescence spectra, probe 14 displayed quenching upon interaction with Hg^2+^ due to photoinduced electron transfer (PET). Conversely, it increased fluorescence when interacting with Sn^2+^, attributed to chelation-enhanced fluorescence (CHEF) (Fig. 13). The sensor demonstrated a limit of detection (LOD) of 1.09 μM for Hg^2+^ and 0.31 μM for Sn^2+^ using fluorometric analysis. The analysis of the Job's plot established a 1 : 2 binding ratio between probe 14 and the Hg^2+^/Sn^2+^ ion. The sensor exhibited exceptional selectivity among competing metal ions and maintained functionality across a broad pH range (3–9).

### 3.2 Rhodamine-BODIPY-based mercury ion sensor

Rhodamine is a family of xanthene-based dyes known for their vibrant fluorescence and excellent photophysical properties. Due to their strong absorption and emission characteristics, these dyes are widely utilized in diverse fields, including water tracing, biotechnology, and materials science. In the realm of sensing, rhodamine-based compounds have gained prominence as chemosensors for detecting ions (*e.g.*, Hg^2+^, Cu^2+^), anions (*e.g.*, CN^−^), and biomolecules (*e.g.*, ATP).^79,80^ These sensors rely on rhodamine's unique “turn-on” fluorescence mechanism triggered by specific interactions with target analytes.

**Scheme 3:**
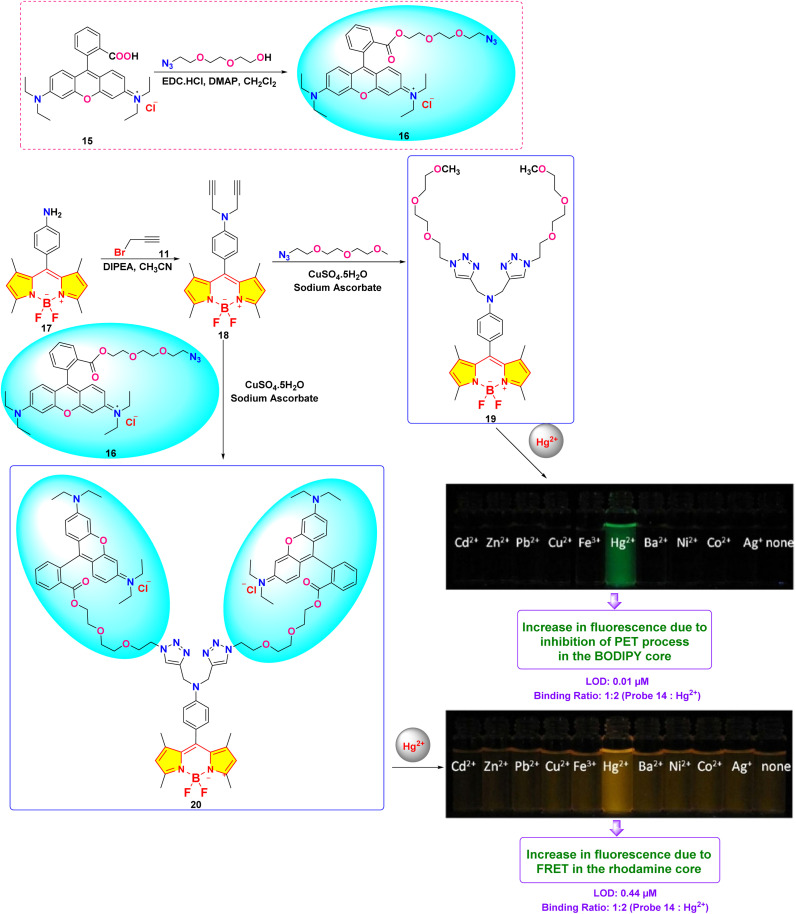
Synthetic route for probes 19 and 20.

**Fig. 14:**
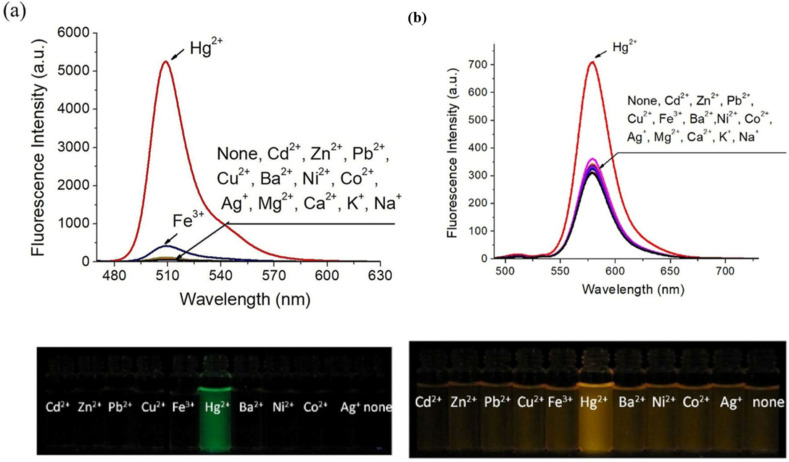
(a) Emission spectra of 19 (2 μm) in CH_3_CN/H_2_O (9 : 1, v/v) upon interaction with different metal ions and the color shifts when exposed to a UV lamp at 365 nm. (b) Emission spectra of 20 (2 μm) in CH_3_CN/H_2_O (9 : 1, v/v) upon interaction with different metal ions and the color shifts when exposed to a UV lamp at 365 nm. Reproduced with permission from ref. 81. Copyright 2019, Wiley-VCH.

In 2019, Shi *et al.* developed two BODIPY-based fluorescent featuring bis(1,2,3-triazole)amino receptors (probe 19) and its rhodamine-substituted analogous (probe 20) for selective Hg^2+^ detection in acetonitrile-aqueous media (9 : 1 v/v) (Scheme 3).^81^ Probe 19 displayed enhanced fluorescence intensity with an 80-fold increase in fluorescence quantum yield upon interaction with 60 equivalent Hg^2+^ ions. Meanwhile, other evaluated metal ions such as Cd^2+^, Zn^2+^, Pb^2+^, Cu^2+^, Ba^2+^, Ni^2+^, Co^2+^, Ag^+^, Mg^2+^, Ca^2+^, K^+^, and Na^+^ did not change the emission spectra significantly. The fluorescence enhancement of probe 19 could be attributed to the inhibition of PET quenching following interaction with Hg^2+^. The rhodamine-substituted analogous (probe 20) displayed similar fluorescence enhancement ascribed to FRET arising from rhodamine moieties (Fig. 14). Job's plot analysis revealed a 1 : 2 binding stoichiometry in CH_3_CN for the interactions between the probes and Hg^2+^ ions, with binding constants determined to be 1.2 ± 0.1 × 10^22^ M^−2^ and 1.3 ± 0.3 × 10^10^ M^−2^ for probes 19 and 20 respectively. The LOD was found to be 0.01 μM for 19 and 0.44 μM for 20. The higher LOD of probe 19 can be attributed to the bulky rhodamine moiety, which may impede efficient analyte binding. Proton NMR titration displayed broadened and downfield shifted triazole proton singlet in probes 19 and 20 due to binding with Hg^2+^ ions.

**Scheme 4:**
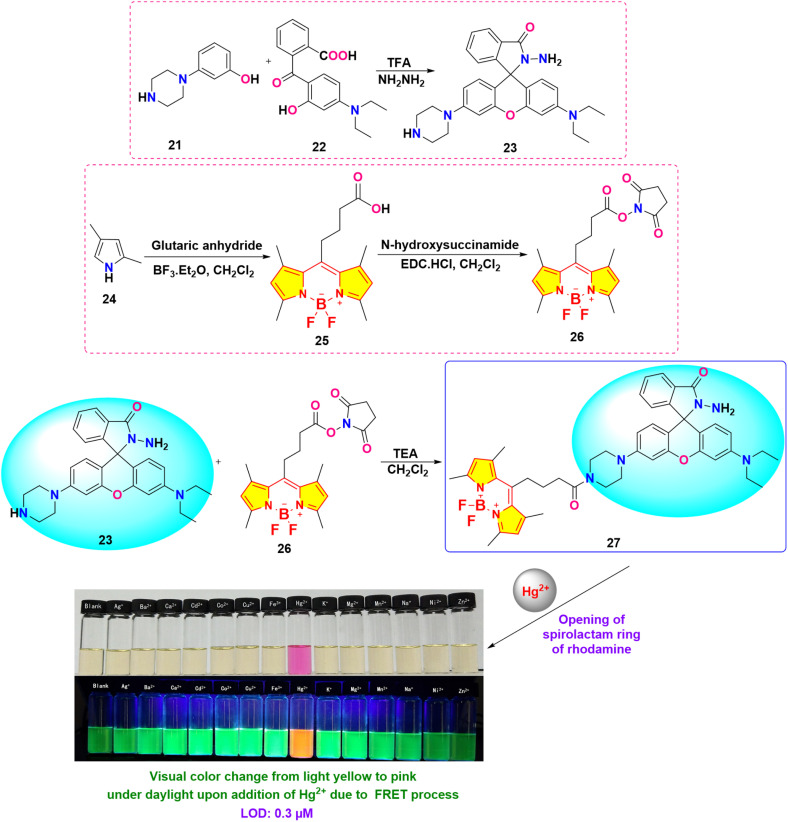
Synthetic route and colorimetric change of probe 27.

**Fig. 15:**
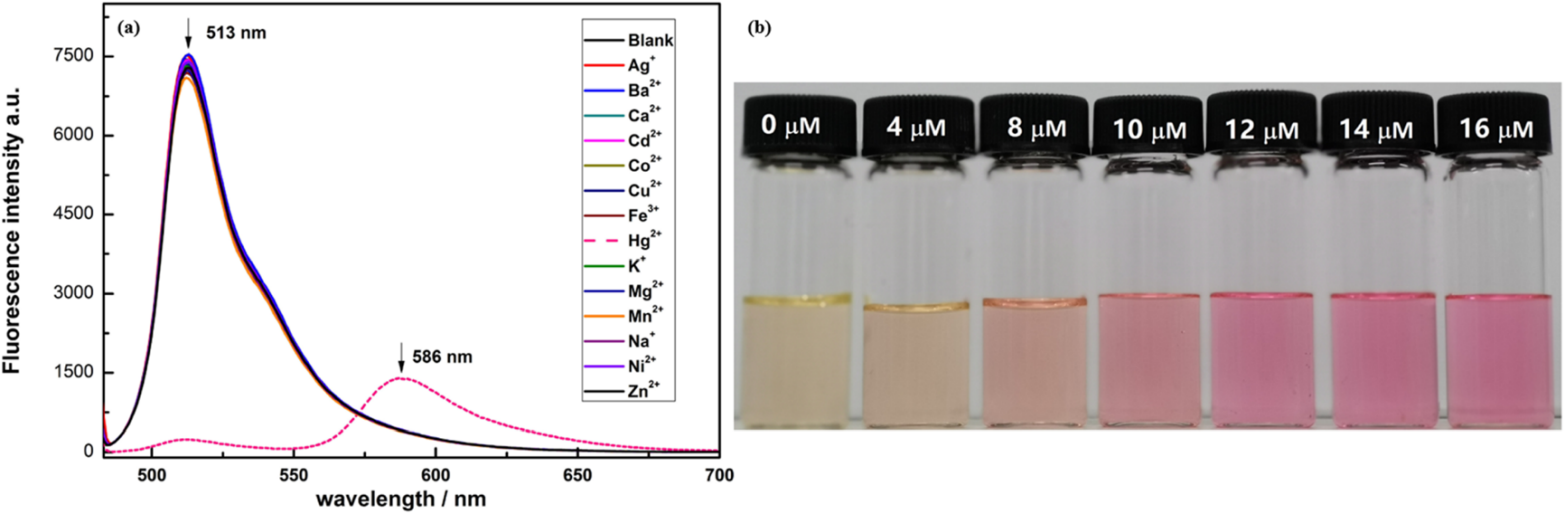
(a) Fluorescence emission of probe 27 (10 μM) with and without various metal ions (100 μM); (b) color change of probe 27 (10 μM) with different concentrations of Hg^2+^ (0–16 μM) in MeOH/20 mM HEPES buffer (V/V = 7 : 3, pH = 6.0) under the daylight. Reproduced with permission from ref. 82. Copyright 2021, Elsevier.

Wen *et al.* developed an innovative BODIPY-Rhodamine-based probe 27 for the selective detection of Hg^2+^ ions, employing a fluorescence resonance energy transfer (FRET) mechanism, with BODIPY serving as the energy donor and Rhodamine functioning as the energy acceptor (Scheme 4).^82^ Spectroscopic measurements were conducted in a 7 : 3 (v/v) mixture of methanol and water at pH 6.0. By the addition of Hg^2+^ ions, a distinct increase in absorbance at approximately 554 nm (characteristic of the Rhodamine moiety) was observed in the UV-vis spectra. This spectral change was accompanied by a visible colorimetric response, with the probe solution changing from yellow to pink under ambient light and from green to orange under UV irradiation. Experimental evidence indicates that Hg^2+^ ions facilitate the conversion of the rhodamine moiety from its spirolactam form to an open-ring structure. Fluorescence studies demonstrated a significant enhancement in emission intensity with the gradual addition of Hg^2+^ ions (Fig. 15). Compared to the blank solution, the fluorescence spectrum of the Hg^2+^-treated probe exhibited a new emission band at 586 nm, while the intensity of the original emission band at 513 nm decreased. This “turn-on” fluorescence response is attributed to the efficient energy transfer from the excited BODIPY donor to the Rhodamine acceptor. The LOD for Hg^2+^ ions using probe 27 was determined to be 0.3 μM. The colorimetric and fluorometric responses showed remarkable selectivity for Hg^2+^ ions, displaying no significant changes with other metal ions present.

### 3.3 Thiosemicarbazide-BODIPY-based mercury ion sensor

Thiosemicarbazide, a versatile organosulfur compound, is widely recognized for its strong ability to coordinate with metal ions thanks to its electron-donating nitrogen and sulfur atoms. This property makes it a valuable reagent in analytical chemistry, particularly for detecting transition and heavy metal ions. Its chemical structure enables it to form stable complexes with metals, facilitating its use in various sensing platforms.^83^ In sensing mercury ions (Hg^2+^), thiosemicarbazide-based compounds have proven highly effective because of their high affinity towards mercury ions and capacity to induce distinct optical or fluorescence changes upon binding.

**Scheme 5:**
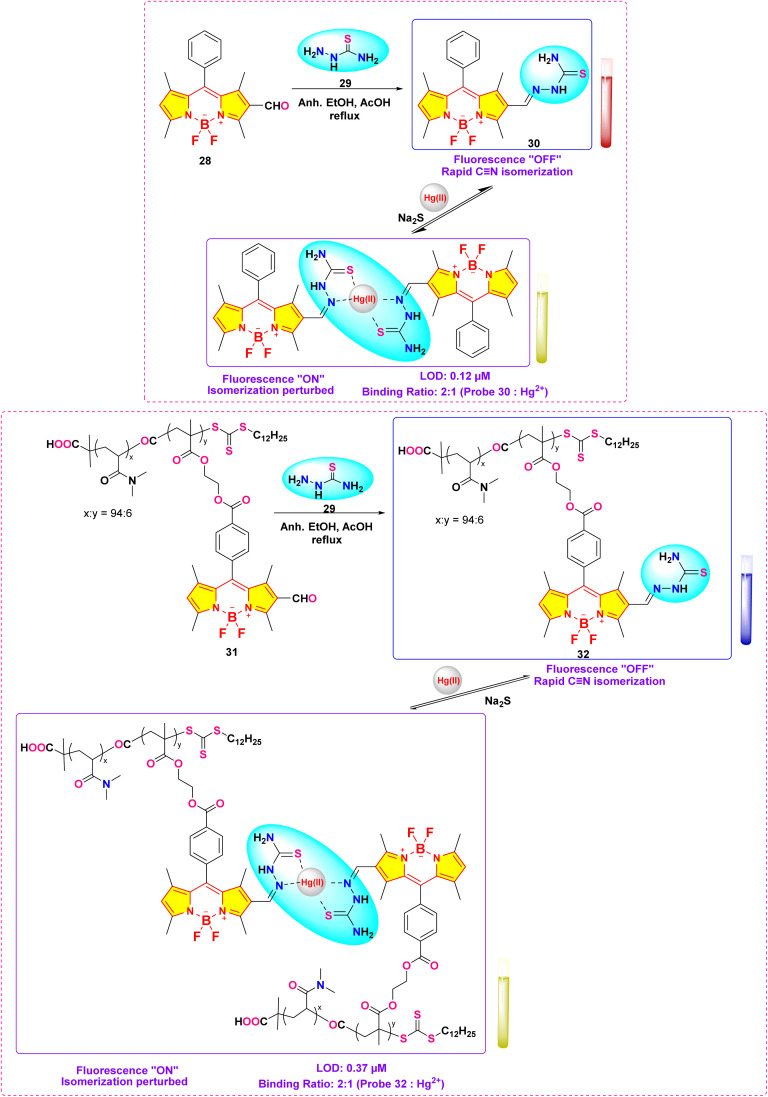
Synthesis and plausible sensing mechanism of probe 32.

In 2019, Haldar and Lee developed a water-soluble polymeric chemosensor (probe 32) integrating BODIPY fluorophores and thiosemicarbazone receptors for selective Hg^2+^ detection and removal in pure aqueous media (Scheme 5).^84^ The copolymer exhibited a turn-on fluorescence response at 545 nm (*Φ*_F_ = 0.17) upon Hg^2+^ coordination, achieving a detection limit of 0.37 μM in HEPES buffer (pH 7.4). Spectroscopic analysis showed that the binding of Hg^2+^ inhibited isomerization at the C=N bond of the thiosemicarbazone group, thereby restoring the inherent fluorescence of BODIPY. Job plot analysis confirmed a 2 : 1 binding stoichiometry between probe 32 and Hg^2+^, with ^1^H NMR indicating upfield shifts of imino and methyne protons upon complexation. The sensor demonstrated exceptional selectivity among 20 competing metal ions and maintained functionality in aqueous solution, addressing hydrolysis issues observed in its small-molecule analog (probe 30). Notably, probe 32 enabled 86% Hg^2+^ removal from contaminated water *via* precipitation of coordination complexes, validated by ICP-OES analysis.

**Scheme 6:**
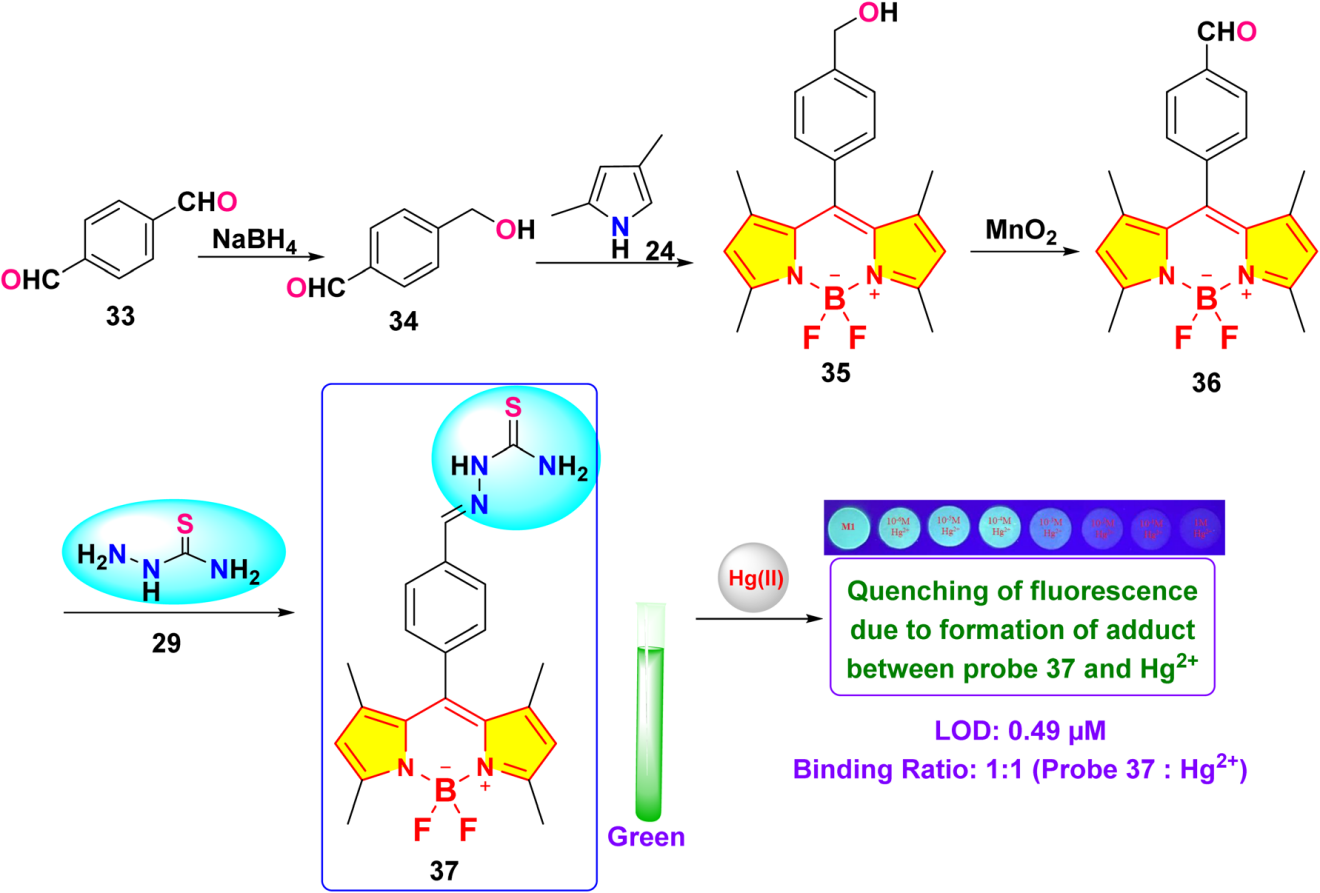
Synthetic route for probe 37.

**Fig. 16:**
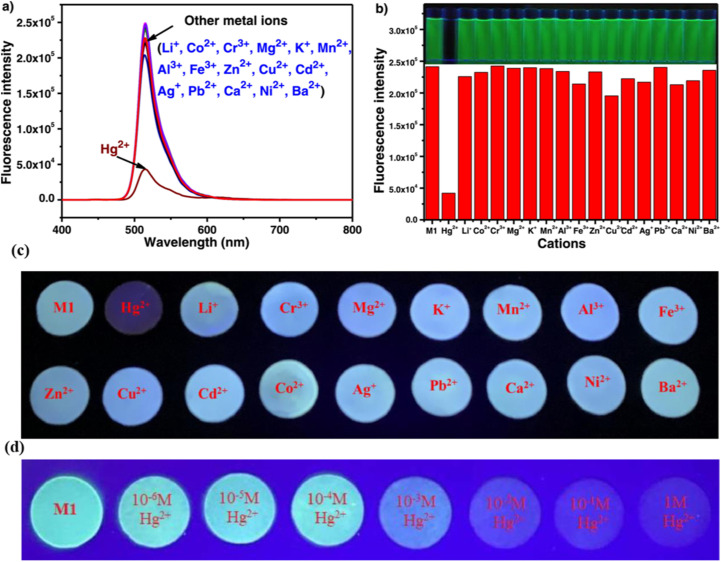
(a) Emission spectra of probe 37 in H_2_O/DMF (8 : 2) upon the addition of 4 equivalents of various metal ions; (b) fluorescence intensity at 517 nm of probe 37 in H_2_O/DMF (8 : 2) with various metal ions (5 equivalents). (c) Test strips exposed to various metal ions when illuminated at 365 nm. (d) Images of the test strips treated with different concentrations of mercury ions. Reproduced with permission from ref. 44. Copyright 2022, Elsevier.

Chen *et al.* reported the development of a novel fluorescence sensor (probe 37) for selective detection of Hg^2+^ ions based on a thiosemicarbazide-functionalized boron dipyrromethene (BODIPY) derivative (Scheme 6).^44^ Spectroscopic investigations were conducted in a binary solvent mixture of water and dimethylformamide (DMF) (8 : 2, v/v). After adding four equivalents of Hg^2+^, probe 37's green emission was significantly quenched, unlike other metal ions (Fig. 16). The fluorescence titration demonstrated a reduction in the intensity of the emission band at 517 nm. The limit of detection was determined to be 0.49 μM, suggesting a 1 : 1 binding stoichiometry between probe 37 and Hg^2+^ ions based on the analysis conducted *via* Job's plot analysis. The association constant for the interaction between probe 37 and Hg^2+^ was determined to be 2.98 × 10^4^ M^−1^. ^1^H NMR titration indicated broadening of the singlet at 11.52 ppm, attributed to N–H, upon the addition of Hg^2+^ ions. To demonstrate practical applicability, a successful paper strip test for Hg^2+^ ion detection was implemented using the synthesized probe.

### 3.4 Styryl-BODIPY-based mercury ion sensor

The styryl group is a conjugated system that plays a crucial role in medicinal chemistry, materials science, and sensing applications.^85–88^ Its extended π-conjugation imparts unique optical and electronic properties, including solvatochromism, fluorescence, and high photostability.^89^ Additionally, the styryl group can be functionalized to enhance its interaction with specific targets, allowing for its integration into various chemical and biological systems. In sensing technologies, styryl-based compounds are widely used as chemosensors to identify metal ions, anions, and biomolecules through resonance energy transfer (RET) or intramolecular charge transfer (ICT).^90^

**Scheme 7:**
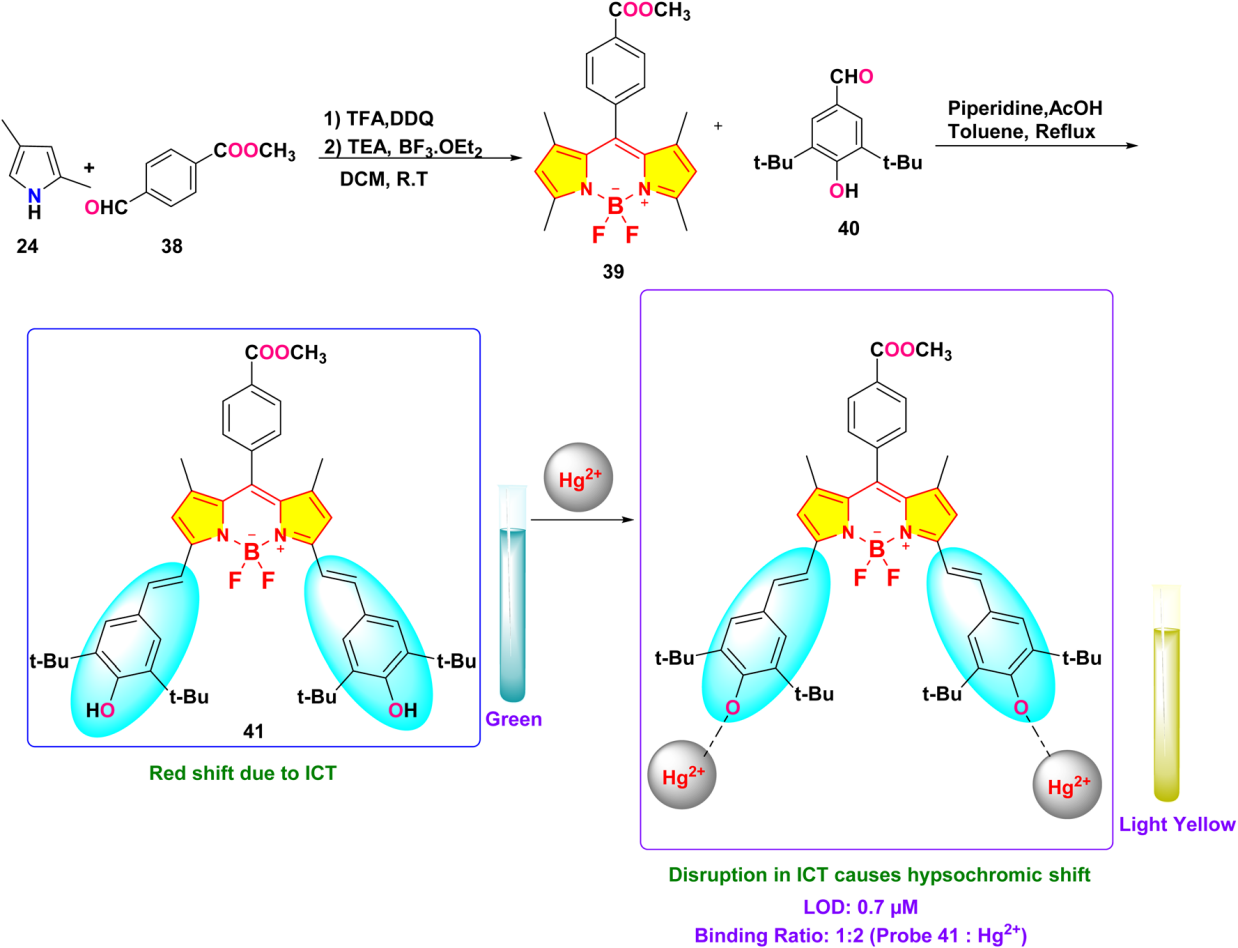
Synthesis and plausible sensing mechanism of probe 41.

**Fig. 17:**
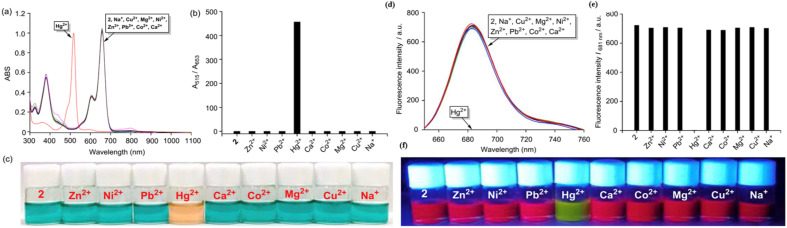
(a) UV-vis spectra of probe 41 upon addition of 5 equivalent of different cations; (b) absorption ratio of (*A*_515_/*A*_653_) of probe 41 containing 50 equivalent of different metals; (c) color change of probe 41 in the presence of different metal ions; (d) fluorescence spectra of probe 41 upon addition of 50 equivalent of different cations; (e) the fluorescence responses at 681 nm (*I*_681_) of probe 41 containing 50 eq. of Hg^2+^ ions introduced to chosen metal ions (50 eq.) in a THF–H_2_O mixture (v/v = 1 : 1; HEPES 10 mM, pH = 7.2) solution; (f) color of solution with different metal ions under UV irradiation at 360 nm. Reproduced with permission from ref. 91. Copyright 2019, Elsevier.

In 2019, Xue *et al.* developed a distyryl BODIPY-based chemosensor (probe 41) for dual-mode detection of Hg^2+^, demonstrating both chromogenic and fluorogenic responses in an aqueous-THF medium (1 : 1 v/v; HEPES buffer, pH 7.2) (Scheme 7).^91^ The probe demonstrated a distinct color transition from green to light yellow (absorption shift: 653 nm → 515 nm) and fluorescence quenching at 681 nm (quantum yield: 0.89 → 0.03) upon Hg^2+^ coordination, enabling naked-eye detection (Fig. 17). Spectroscopic analyses revealed a Hg^2+^-induced blocking of the intramolecular charge transfer (ICT) process from hydroxyl-substituted distyryl groups to the BODIPY core observed, with a detection limit of 0.7 μM calculated *via* fluorometric titration. Job plot and ^1^H NMR studies confirmed a 1 : 2 binding stoichiometry (probe 41: Hg^2+^), where Hg^2+^ initially oxidizes hydroxyl groups to quinone intermediates before coordinating with the phenolic oxygen atoms. The sensor exhibited exceptional selectivity among 10 competing metal ions, including Pb^2+^ and Cu^2+^, with minimal interference. Unique among Hg^2+^ probes, probe 41 utilized hard donor hydroxyl receptors rather than conventional soft sulfur-based ligands, achieving a binding constant of 4.3 × 10^3^ M^−1^.

**Scheme 8:**
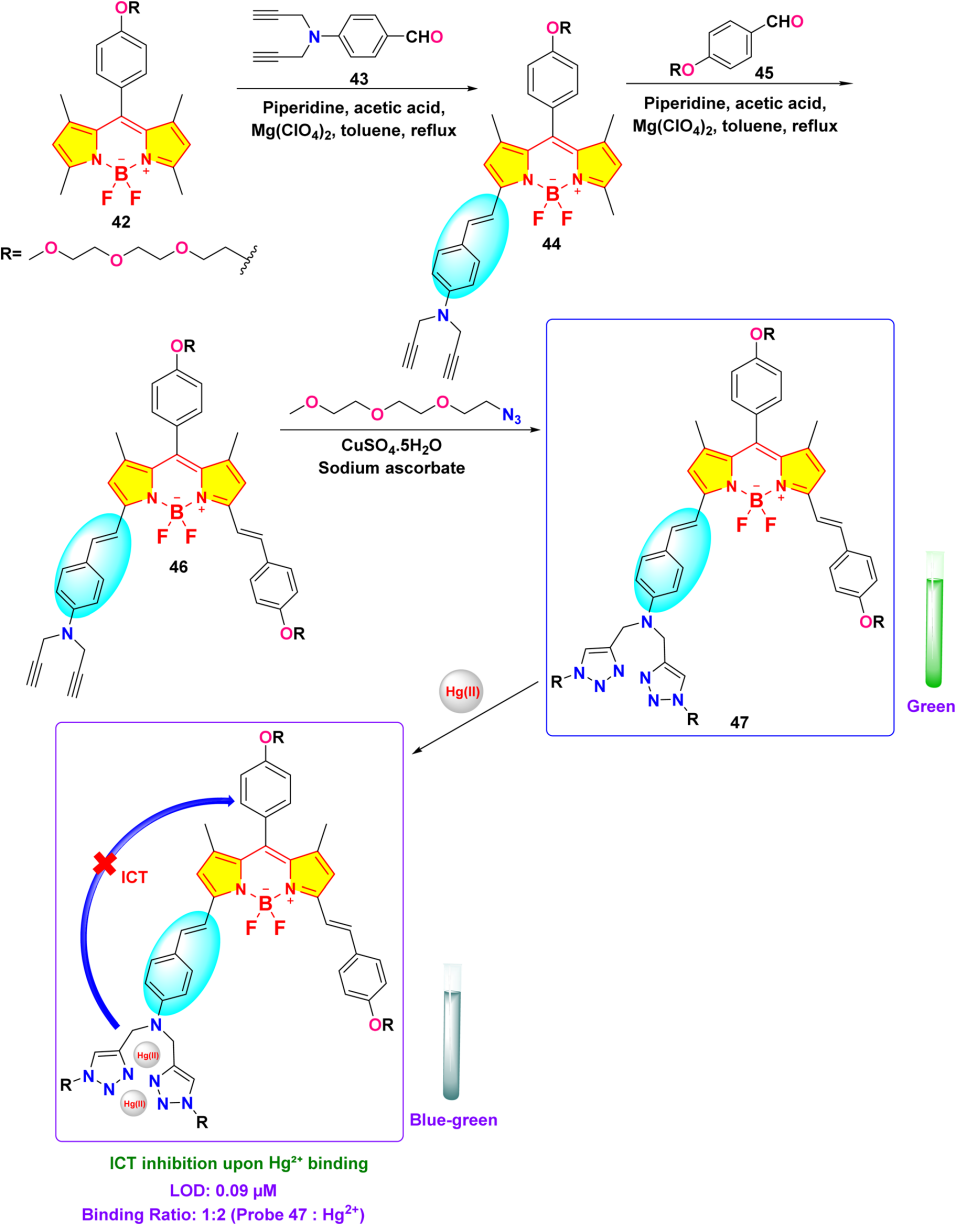
Synthesis and plausible sensing mechanism of probe 47.

**Fig. 18:**
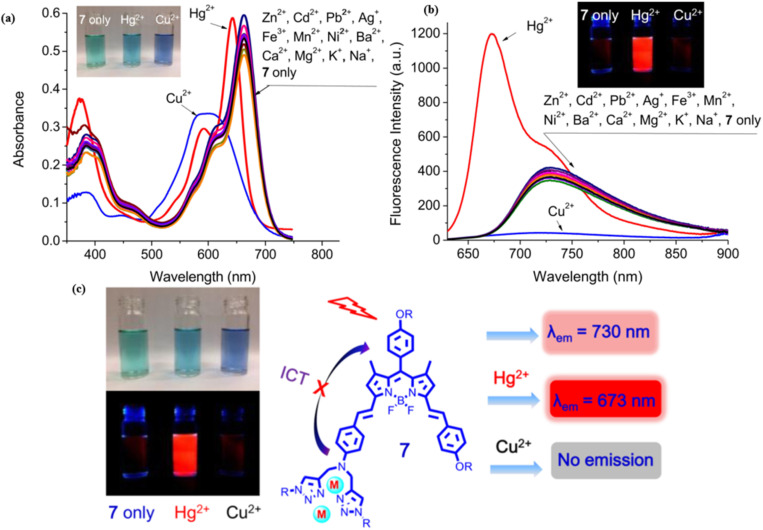
(a) Change in absorption spectra of probe 47 (6 μM) in CH_3_CN/H_2_O (5 : 1 v/v) upon addition of different metal ions (20 equiv.); (b) change in fluorescence spectra of probe 47 (6 μM) in CH_3_CN/H_2_O (5 : 1 v/v) upon addition of different metal ions (20 equiv.); (c) the ratiometric and discriminative detection of Hg^2+^ and Cu^2+^ ions; Reproduced with permission from ref. 92. Copyright 2018, Elsevier.

In 2019, Huang *et al.* developed a near-infrared distyryl BODIPY-based chemosensor (probe 47) functionalized with a bis(1,2,3-triazole)amino receptor for quick and selective detection of Hg^2+^ and Cu^2+^ in CH_3_CN/H_2_O (5 : 1 v/v) (Scheme 8).^92^ The probe 47 demonstrated notable spectral responses upon interaction with Hg^2+^ and Cu^2+^ but did not display any changes upon interaction with other metals such as Ni^2+^, Mn^2+^, Zn^2+^, Fe^3+^, Co^2+^, Cd^2+^, Pb^2+^, Ba^2+^, Ag^+^, K^+^, Mg^2+^, Na^+^, Pd^2+^, and Ca^2+^. Hg^2+^ induced a blue shift of 22 nm in absorption (from 663 nm to 641 nm) and exhibited ratiometric fluorescence enhancement at 673 nm (detection limit: 0.09 μM). In contrast, Cu^2+^ resulted in absorption broadening (from 663 nm to 597 nm) and fluorescence quenching at 730 nm (detection limit: 1.02 μM) (Fig. 18). Binding studies revealed a 1 : 2 stoichiometry (probe 47: Hg^2+^/Cu^2+^) for both ions through Job plot analysis, with binding constants of 1.0 × 10^9^ M^−2^ (Hg^2+^) and 2.0 × 10^10^ M^−2^ (Cu^2+^), indicating stronger coordination with Cu^2+^. The response to Hg^2+^ was attributed to intramolecular charge transfer (ICT) inhibition, whereas Cu^2+^ prompted the quenching of photoinduced electron transfer (PET). The study demonstrated exceptional selectivity among 20 competing metal ions, including Pb^2+^ and Fe^3+^, with rapid response kinetics of less than five minutes. Cellular imaging conducted in HeLa cells confirmed dual-channel discrimination, indicating a 14-fold increase in the *I*_640–700_/*I*_701–758_ ratio for Hg^2+^ as opposed to complete quenching observed for Cu^2+^.

### 3.5 Pyrrolyl-BODIPY-based mercury ion sensor

The pyrrolyl group, a structural unit derived from pyrrole (C_4_H_4_NH), is a five-membered aromatic heterocycle that contains a nitrogen atom in its conjugated π-electron system. This electron-rich structure, characterized by the partial delocalization of the nitrogen lone pair into the ring, facilitates strong coordination with metal ions and enhances charge–transfer interactions. These properties make the pyrrolyl group a versatile building block in developing chemosensors for environmental, biomedical, and industrial applications.

**Scheme 9:**
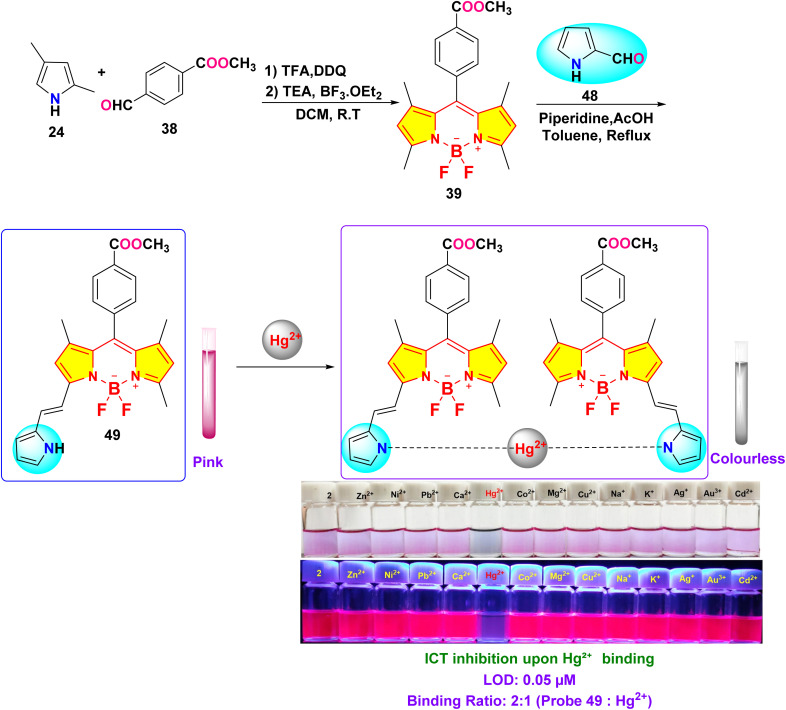
Synthesis and plausible sensing mechanism of probe 49.

**Fig. 19:**
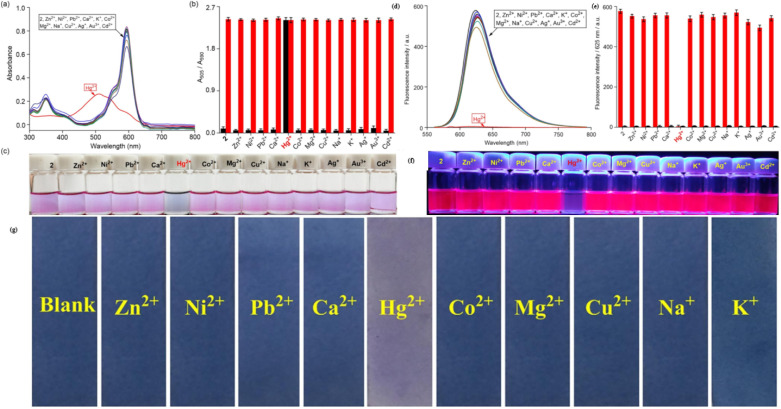
(a) Change of UV-vis absorption spectra of 49 (*c* = 5.5 μM) mixed with different metal cations (25 eq.). (b) The absorbance ratios between *A*_505_/*A*_591_ responses of chemosensor 49 containing of 25 eq. Hg^2+^ ion to the selected different kinds of cations (25 eq.). (c) Changes of the solution color of 49 mixed with different metal cations. (d) Emission spectra (excitation at 587 nm) of 49 (*c* = 5.5 μM) measured in a THF–H_2_O solution (v/v = 1 : 1; HEPES 10 mM, pH = 7.2) with the presence of Hg^2+^ ion and other metal ions (25 eq.). (e) Fluorescence intensity at 625 nm (*I*_625_) of probe 49 before and after the addition of 25 eq. of Hg^2+^ ion and other metal cations solution (25 eq.); (f) photos of solution colors for 49 in the presence of different metal cations illuminated with UV lamp at 360 nm (g) photographs of the probe 49 based test strips colorimetric detect different cations. Reproduced with permission from ref. 93. Copyright 2021, Elsevier.

In 2022, Chan *et al.* developed a mono-pyrrolyl substituted BODIPY chemosensor (probe 49) for ratiometric Hg^2+^ detection in aqueous-THF media (1 : 1 v/v; HEPES buffer, pH 7.2) (Scheme 9).^93^ The sensor exhibited a distinct colorimetric response from pink to colorless alongside fluorescence quenching at 625 nm (quantum yield: 0.51 → 0) *via* Hg^2+^-induced perturbation of the intramolecular charge transfer (ICT) process between the pyrrole donor and BODIPY core (Fig. 19). Spectrophotometric analyses revealed hyperchromic absorption shifts at 505 nm and 346 nm with a detection limit of 0.05 μM, supported by a binding constant of 5.2 × 10^3^ M^−1^ derived from Benesi–Hildebrand plots. Probe 49 demonstrated exceptional selectivity among 14 competing metal ions, including Pb^2+^ and Cu^2+^, with stable performance across pH 4–9. Job plot and ^1^H NMR titrations confirmed a 1 : 2 binding stoichiometry, where Hg^2+^ coordinates to pyrrole nitrogen atoms, evidenced by NH proton signal disappearance at 8.87 ppm. Practical utility was validated through paper test strips showing visible color transitions for Hg^2+^ concentrations ≥1 μM.

### 3.6 Catechol-BODIPY-based mercury ion sensor

Catechol, also known as pyrocatechol or 1,2-dihydroxybenzene, is a versatile organic compound with a broad array of biological and chemical applications. Its structure, featuring two hydroxyl groups attached to a benzene ring in an *ortho* configuration, imparts unique redox properties and strong metal ion chelating abilities.^94,95^ These characteristics make catechol an essential building block in various fields, including agrochemicals, pharmaceuticals, and materials science.^96–98^ Catechol is particularly valuable in sensing applications because it can form stable complexes with metal ions and undergo oxidation–reduction reactions, which can generate detectable optical or electrochemical signals.

**Scheme 10:**
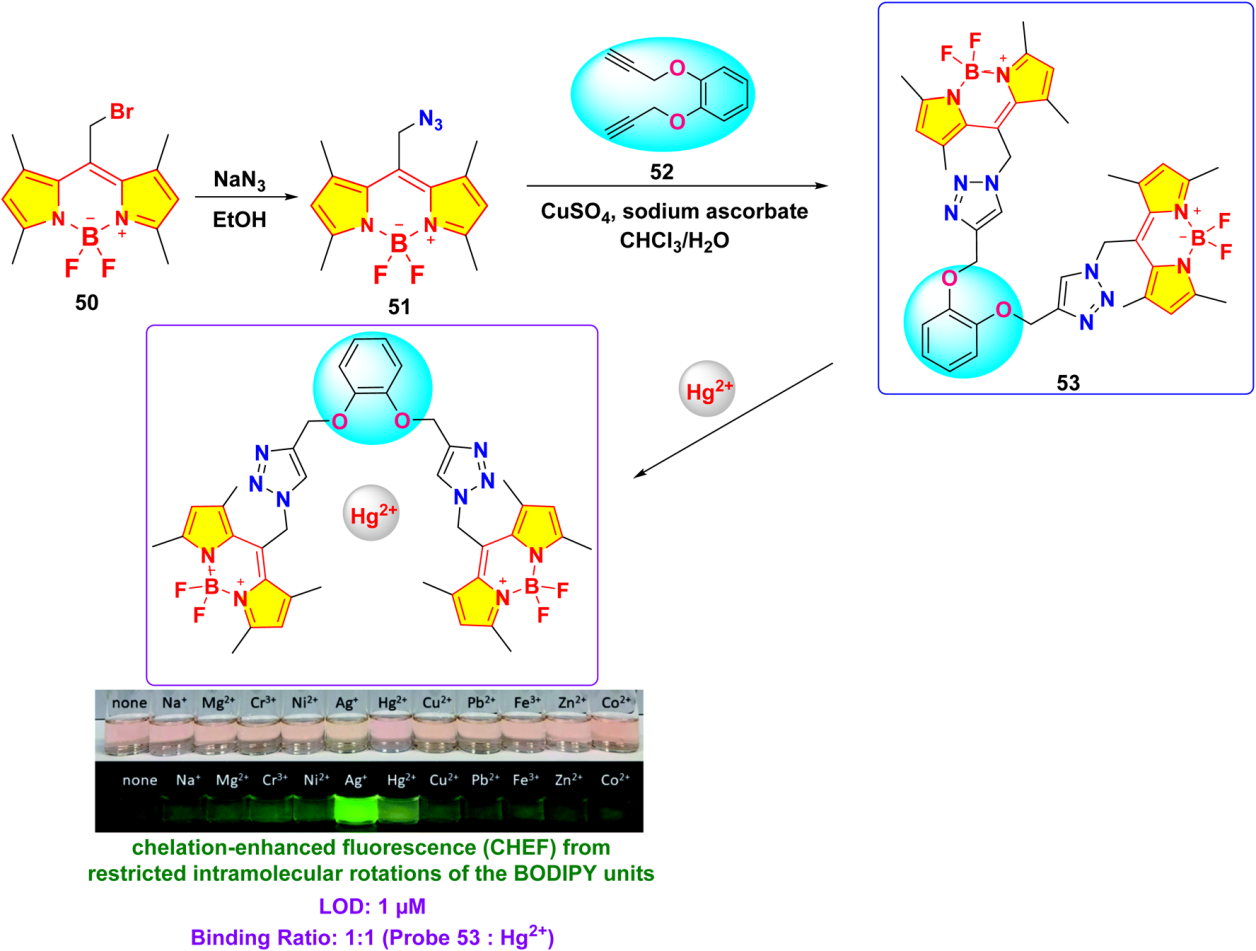
Synthesis and plausible sensing mechanism of probe 53.

**Fig. 20:**
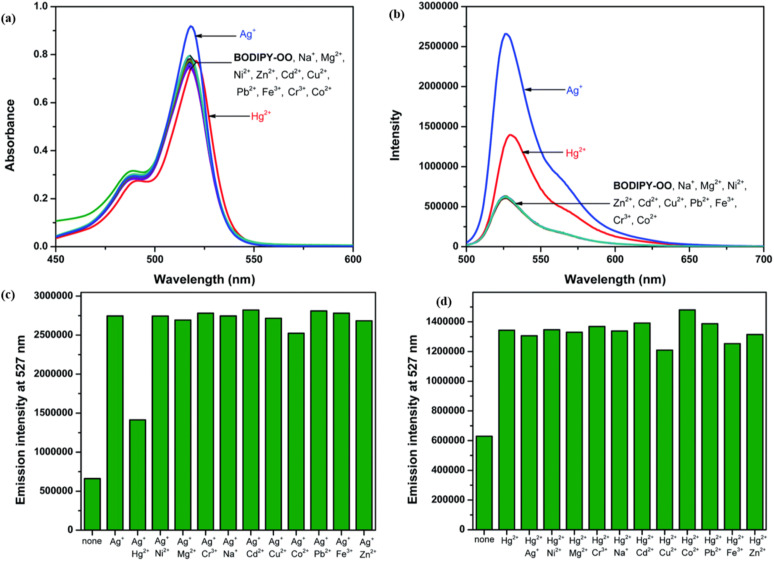
(a) Absorption spectra of probe 53 (5 mM) in the presence of various metal ions (20 equiv.) in MeOH. (b) Emission spectra of probe 53 (0.5 mM) in the presence of various metal ions (20 equiv.) in MeOH (c) competitive selectivity of probe 53 (0.5 mM) toward Ag^+^ (10 equiv.) with other metal cations, and (d) probe 53 (0.5 mM) toward Hg^2+^ (10 equiv.) with other metal cations. Reproduced with permission from ref. 99. Copyright 2021, Elsevier.

In 2021, Saiyasombat and Kiatisevi developed a bis-BODIPY-linked triazole chemosensor (probe 53) anchored on a catechol core for dual detection of Ag^+^ and Hg^2+^ in methanol media (Scheme 10).^99^ The probe exhibited a detection limit (LOD) of 1 μM for Hg^2+^ ions. Upon the addition of Hg^2+^ ions in methanol, the UV-visible absorption spectra displayed a slight red shift, accompanied by a hyperchromic shift in fluorescence spectra (Fig. 20). The increase in fluorescence intensity is due to the chelation-enhanced fluorescence (CHEF) effect, which occurs when intramolecular rotations of the BODIPY fluorophore are restricted after it complexes with Hg^2+^ ions. Job's plot analysis indicated a 1 : 1 binding stoichiometry between probe 53 and Hg^2+^ ions. The probe's binding affinity for Hg^2+^ ions was measured at 1.41 × 10^5^ M^−1^ through the Benesi–Hildebrand equation.

### 3.7 Dithia-azacrown-BODIPY-based mercury ion sensor

Dithia-azacrown ethers represent a group of macrocyclic compounds characterized by the incorporation of sulfur (thioether) and nitrogen (aza) donor atoms within their cyclic framework. This unique combination of heteroatoms imparts exceptional binding properties towards specific metal ions, making dithia-azacrown ethers highly valuable in supramolecular chemistry and sensing applications. Their ability to create stable complexes with transition and heavy metal ions arises from the synergistic interaction between the soft sulfur donors and the nitrogen atoms, which provide complementary coordination environments. These compounds are also highly tunable, allowing for modifications that optimize their selectivity and sensitivity for targeted analytes.

**Scheme 11:**
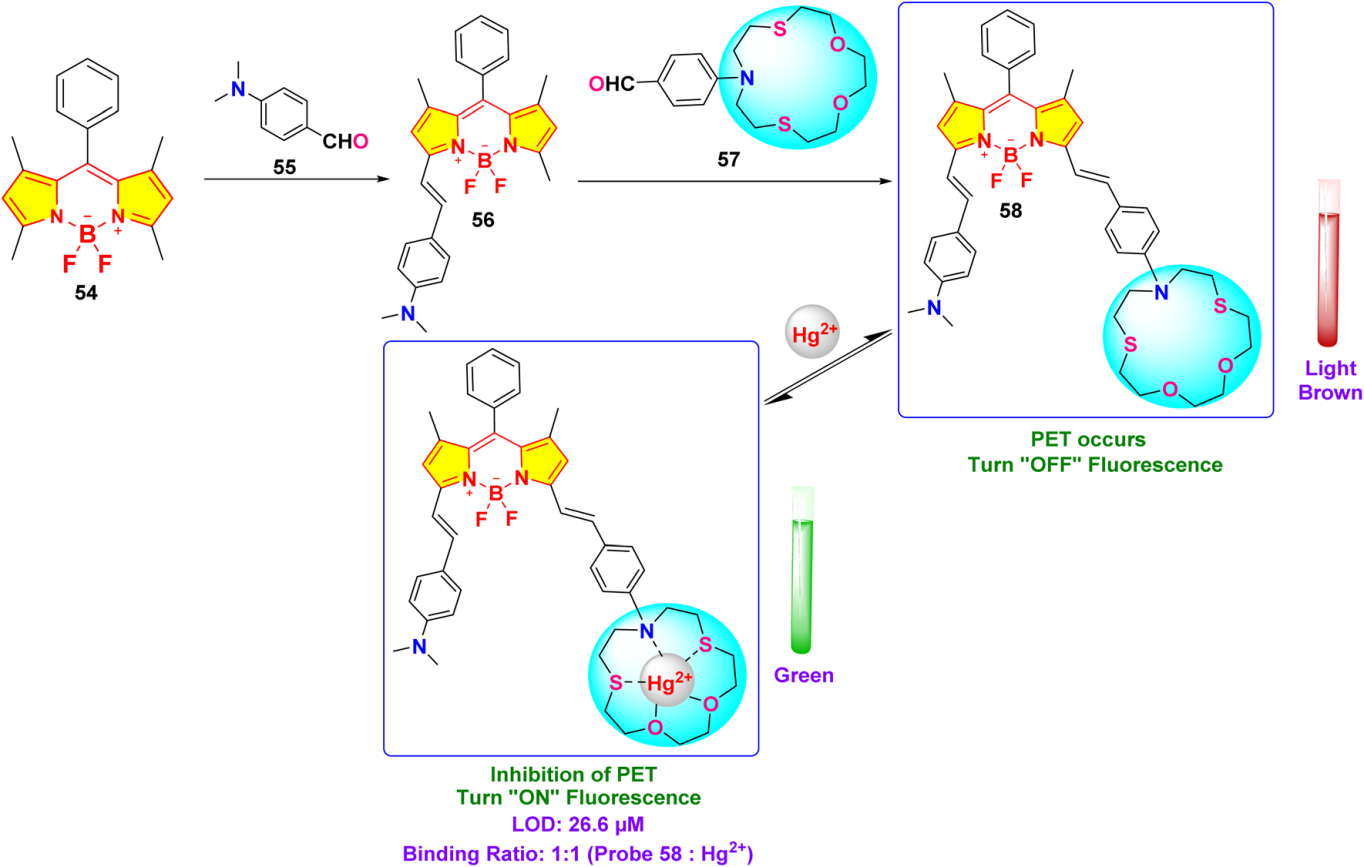
Synthesis and plausible sensing mechanism of probe 58.

**Fig. 21:**
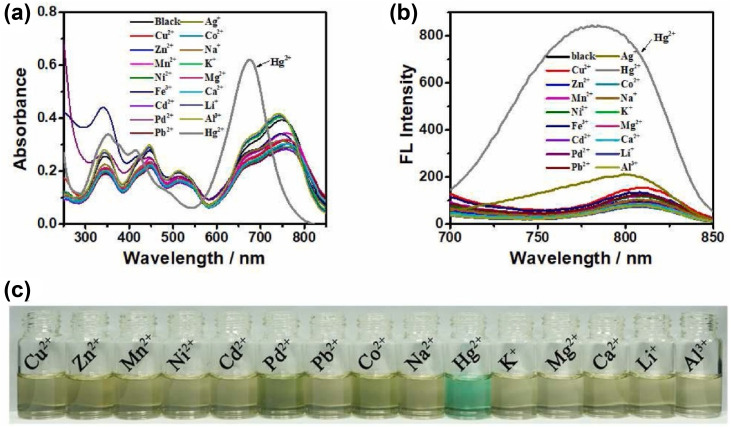
(a) UV-vis spectra of probe 58 in presence of different ions. (b) Emission spectra of probe 58 in presence of different ions. (c) Color change of probe 58 in daylight upon addition of 100 μM of different ions in DMF–H_2_O (v/v = 3 : 7). Reproduced with permission from ref. 100. Copyright 2022, Elsevier.

Chen and colleagues created a fluorescent probe based on near-infrared (NIR) BODIPY (probe 58) featuring a macrocyclic thioether receptor for selective Hg^2+^ detection across biological and environmental systems (Scheme 11).^100^ Upon the addition of Hg^2+^ ions in a DMF–water (3 : 7, v/v) solution, the UV-vis absorption spectrum of probe 58 exhibited a significant spectral change: the original absorption band in the 580–820 nm region disappeared, while a new intense peak emerged at 670 nm. The presence of an isobestic point at 701 nm further supports the specific interaction between the probe and Hg^2+^ ions. Concurrently, a distinct colorimetric change from light brown to green was observed, indicating the probe's suitability for colorimetric sensing of Hg^2+^ ions. Fluorescence studies indicated a modest red shift in the emission peak, shifting from 809 nm to 790 nm, with a significant increase in fluorescence intensity following the introduction of Hg^2+^ ions. The exciting “turn-on” fluorescence response occurs when photoinduced electron transfer (PET) from the ligand to the BODIPY core is inhibited after the formation of the Hg^2+^–probe complex (Fig. 21). We found that the binding constant between probe 39 and Hg^2+^ ions is a strong 6.4 × 10^4^ M^−1^, with a LOD calculated at 26.6 μM. Job's plot analysis indicated a 1 : 1 binding stoichiometry between the probe and Hg^2+^ ions, implying that a stable 1 : 1 complex is likely formed through the fitting of the Hg^2+^ ion within the probe's macrocyclic ring.

### 3.8 Chitosan-BODIPY-based mercury ion sensor

Chitosan, a natural polysaccharide derived from chitin, is a highly versatile biopolymer known for its biocompatibility, biodegradability, non-toxicity, and antimicrobial properties. These characteristics have made it a valuable material in various fields, including medicine, food safety, environmental monitoring, and water treatment.

**Scheme 12:**
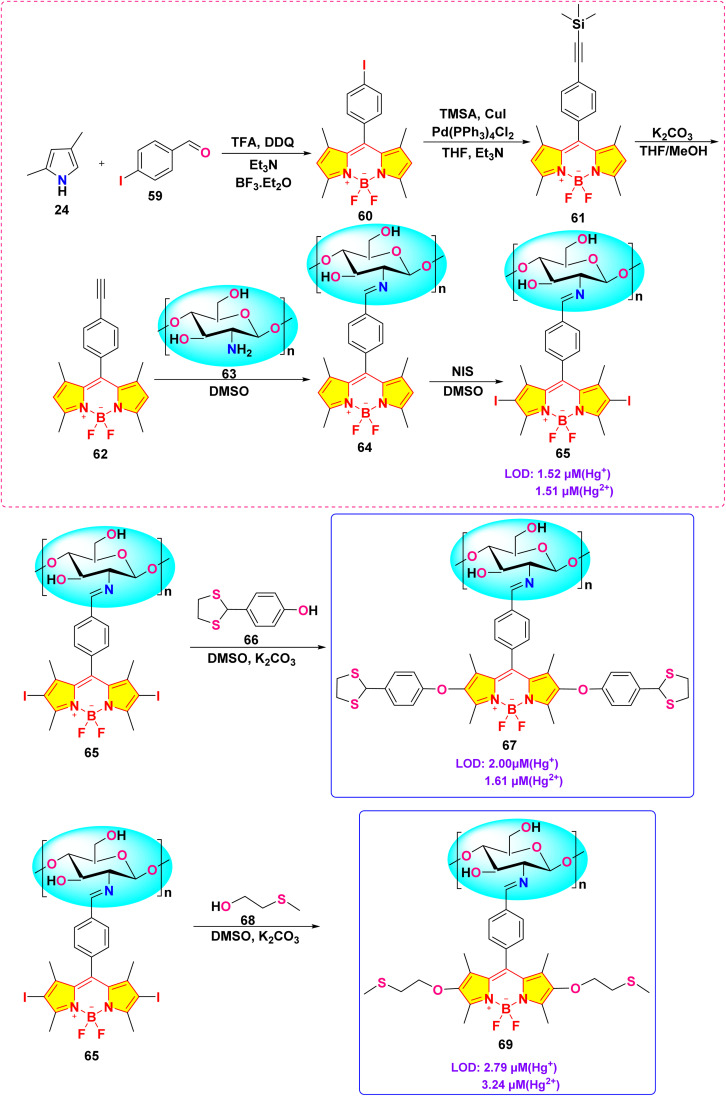
Synthetic route for probe 65, 67 and 69.

Wang *et al.* developed a novel chitosan-based BODIPY macromolecular chemosensor (probe 65, 67 and 69) for the selective detection of mercury ions (Scheme 12).^101^ The initial chemosensor, probe 65, utilized a –C=N bond formed *via* click chemistry as the primary recognition site for mercury ions. To enhance the sensing capabilities, probes 67 and 69 were subsequently synthesized by incorporating additional recognition sites into the chitosan backbone. These modified chemosensors demonstrated the ability to detect not only mercury ions but also iron ions (Fe^2+^ and Fe^3+^). In the absence of sulfur-containing moieties (as in probe 65), the chemosensor selectively recognized Hg^2+^ and Hg^+^ ions through the cleavage of the –C=N bond. Conversely, the incorporation of sulfur atoms (as in probes 67 and 69) facilitated the recognition of Fe^3+^ and Fe^2+^ ions, presumably through the formation of metal–sulfur bonds. These chemosensors exhibited a significant quenching effect in an acetic acid/water (1 : 99, v/v) solution, easily seen both by the naked eye and with UV light, due to the breaking of the –C=N bond when metal ions are present binding. Notably, the quenching effect was slightly more pronounced for Hg^+^ ions compared to Hg^2+^ ions. The cleavage of the –C=N bond was further confirmed by Fourier Transform Infrared (FTIR) spectroscopy. Among probes 67 and 69, probe 67 demonstrated superior sensitivity towards both Hg^2+^ and Hg^+^ ions. The limit of detection (LOD) for these chemosensors was found to be 1.51 μM and 1.52 μM for Hg^2+^ and Hg^+^, respectively, for probe 67; 1.61 μM and 2.00 μM for probe 67; and 3.24 μM and 2.79 μM for probe 69.

**Scheme 13:**
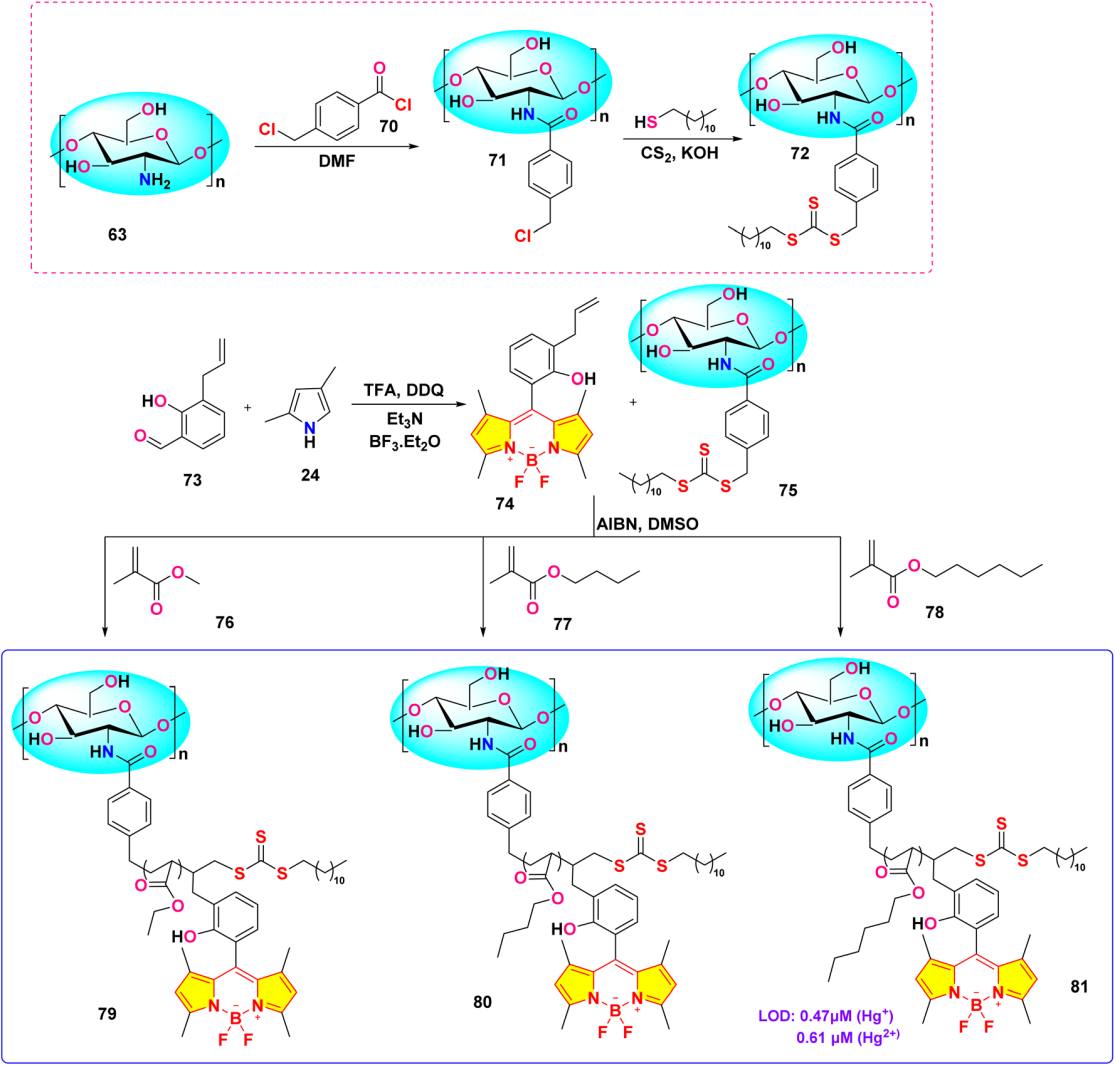
Synthetic route for probe 79, 80 and 81.

**Fig. 22:**
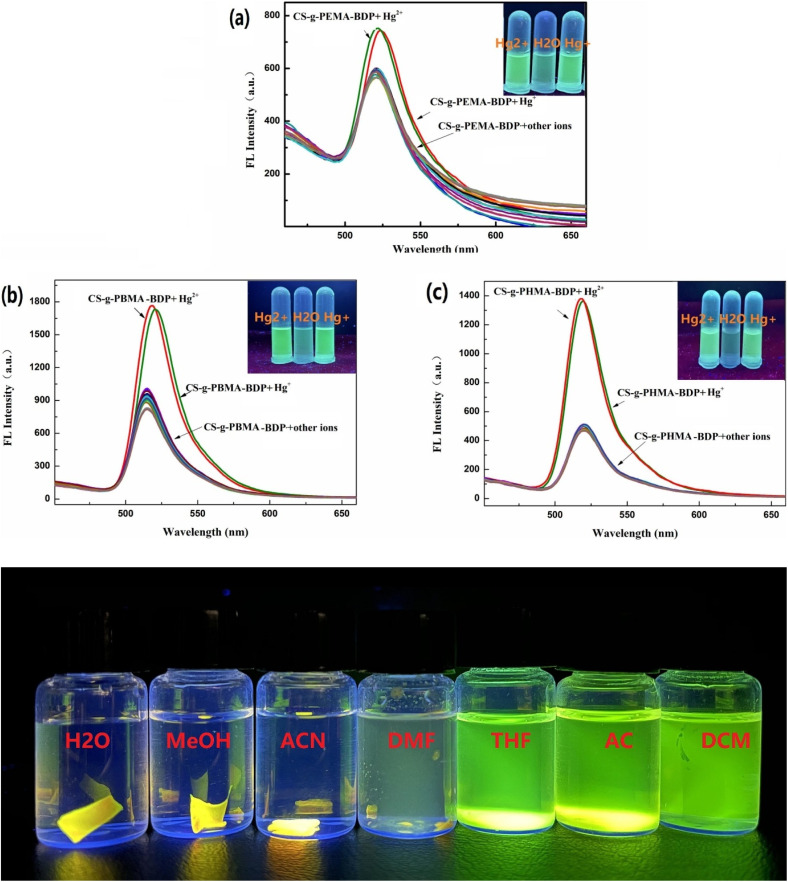
The fluorescent intensity of (a) 79, (b) 80 and (c) 81; and solubility of probe 81 is shown below. Reproduced with permission from ref. 102. Copyright 2018, Elsevier.

In 2023, He *et al.* developed three chitosan-grafted fluorescent probes (probe 79, 80 and 81) *via* RAFT polymerization for selective Hg^2+^/Hg^+^ detection in organic media (Scheme 13).^102^ The probes exhibited “turn-on” fluorescence at 521 nm upon Hg^2+^/Hg^+^ coordination, with probe 81 showing the highest sensitivity and detection limits of 0.61 μM for Hg^2+^ and 0.47 μM for Hg^+^ in DMF (Fig. 22). Spectroscopic analyses revealed a photoinduced electron transfer (PET) inhibition mechanism through Hg-induced coordination to phenolic hydroxyl groups on the BODIPY fluorophore, supported by ^1^H NMR peak shifts at 5.56 ppm and FT-IR C–O bond attenuation at 1270 cm^−1^. The sensors demonstrated exceptional selectivity among the competing metal ions. Practical applications included flexible films and test strips for visual mercury ion detection.

**Scheme 14:**
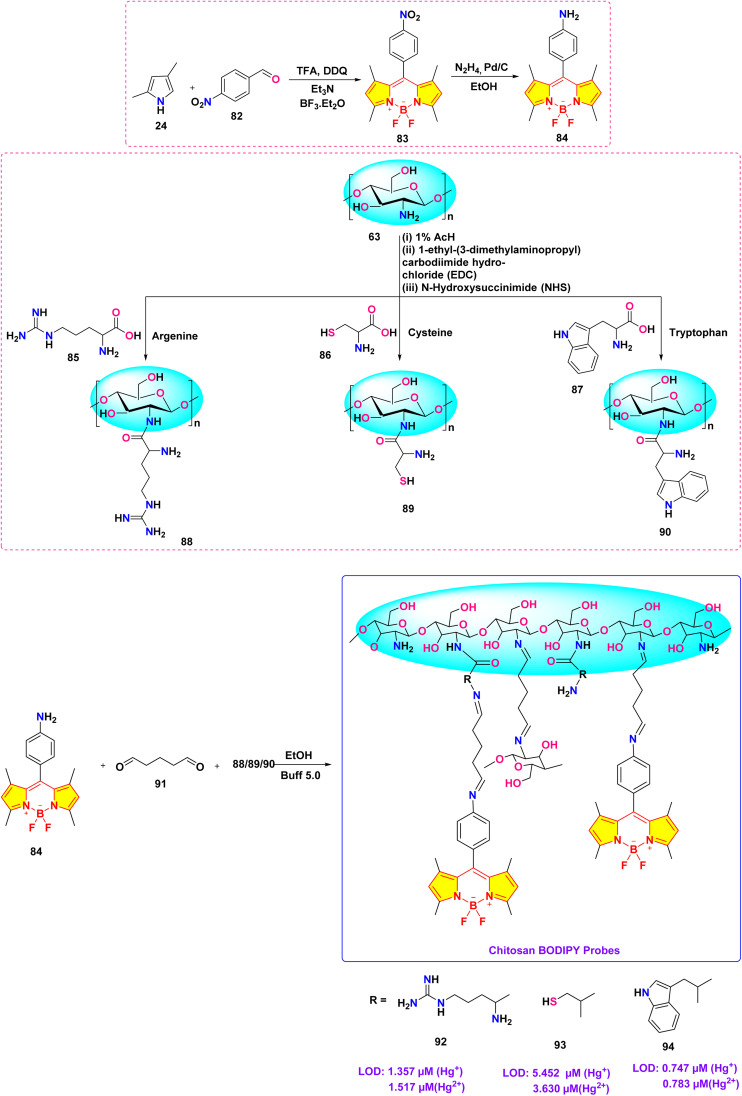
Synthetic route for probe 92, 93 and 94.

**Fig. 23:**
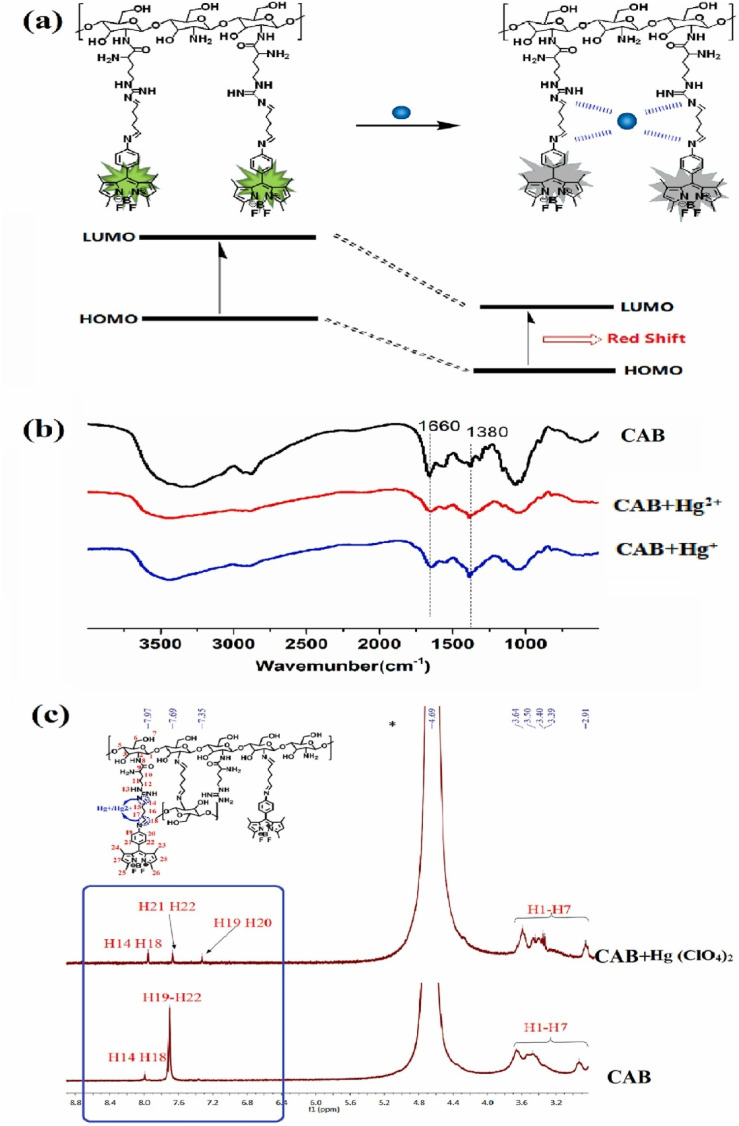
(a) Plausible binding mechanism of probe 92, 93, and 94 (b)FTIR spectra of 92, 92 + Hg^+^ and 92 + Hg^2+^; (c) ^1^H NMR of 92 and 92 + Hg (ClO_4_)_2_. Reproduced with permission from ref. 103. Copyright 2022, Elsevier.

He *et al.* synthesized a series of chitosan-amino acid conjugated BODIPY fluorophores (probe 92, 93 and 94) for the selective detection of Hg^+^ and Hg^2+^ ions (Scheme 14).^103^ Spectroscopic characterization was performed in a 1% acetic acid (HAc) solution. The presence of Hg^+^ and Hg^2+^ ions led to a marked decrease in fluorescence intensity, which is linked to the disruption of intramolecular charge transfer (ICT) processes in BODIPY probes (Fig. 23). The limit of detection for Hg^+^ ions was determined to be 1.357 μM, 5.452 μM, and 0.747 μM, respectively, for probes 92, 93, and 94. Similarly, the LOD for Hg^2+^ ions was found to be 1.517 μM, 3.630 μM, and 0.783 μM for probes 92, 93, and 94, respectively.

### 3.9 Quinoline-BODIPY-based mercury ion sensor

**Scheme 15:**
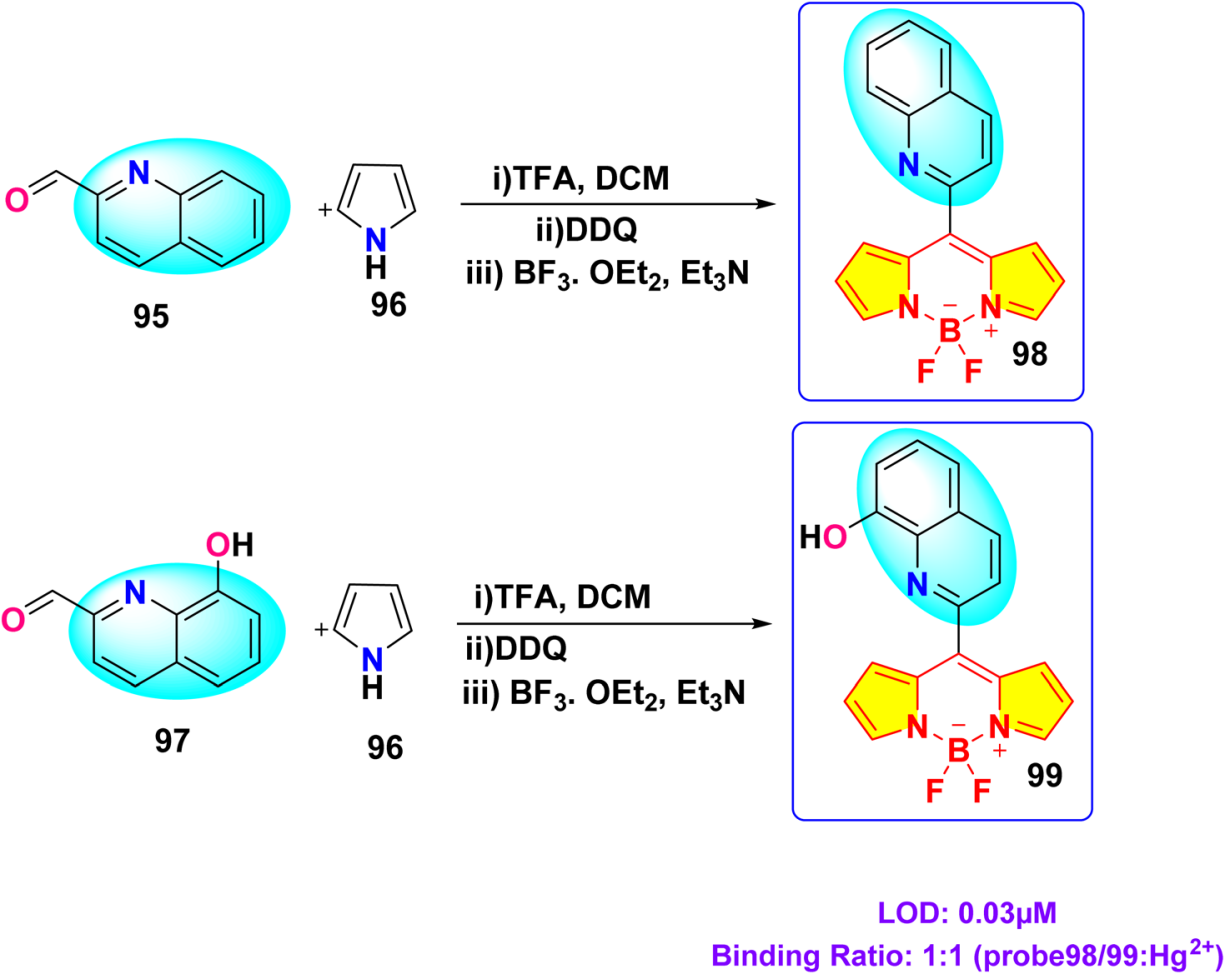
Synthetic route for probes 97 and 99.

**Fig. 24:**
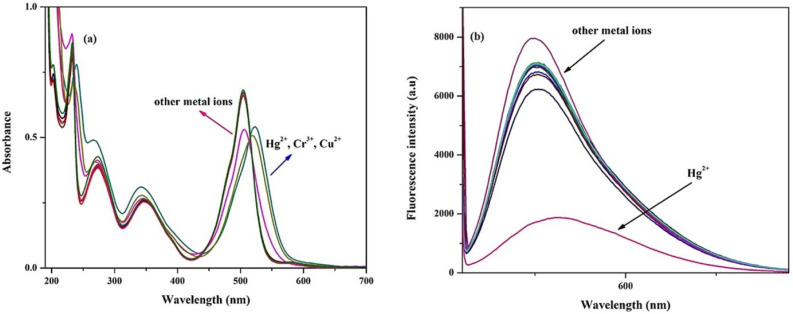
(a) Absorption and (b) emission spectra of probe 97 in acetonitrile/water (7 : 3, v/v) solution with 10 equivalents of various metal ions. Reproduced with permission from ref. 104. Copyright 2024, Elsevier.

In 2024, Kumarasamy *et al.* reported the synthesis of novel quinoline-conjugated BODIPY derivatives (probe 97 and 99) for the selective detection of Hg^2+^ ions in a 7 : 3 (v/v) acetonitrile–water mixture (Scheme 15).^104^ Upon the addition of Hg^2+^ ions, probe 97 exhibited a bathochromic shift in the absorption peak from 505 nm to 510 nm (Fig. 24). Concurrently, the emission spectrum displayed a notable decrease in the fluorescence intensity at 550 nm. The quenching effect occurs due to a photoinduced electron transfer (PET) process from the quinoline group to the BODIPY core, resulting in enhanced fluorescence quenching through chelation. Probe 99 exhibited analogous spectral behavior. Job's plot analysis revealed binding constants of 2.16 × 10^−5^ M^−1^ and 3.27 × 10^−5^ M^−1^ for probe 97 and probe 99, respectively. The probes showed a notable and precise reduction in fluorescence upon exposure to Hg^2+^ ions, achieving an impressively low detection limit of approximately 3.06 × 10^−8^ M. Additionally, Job's plot validated a 1 : 1 binding ratio between the chemosensors and Hg^2+^ ions.

### 3.10 Oxadiazol-BODIPY-based mercury ion sensor

**Scheme 16:**
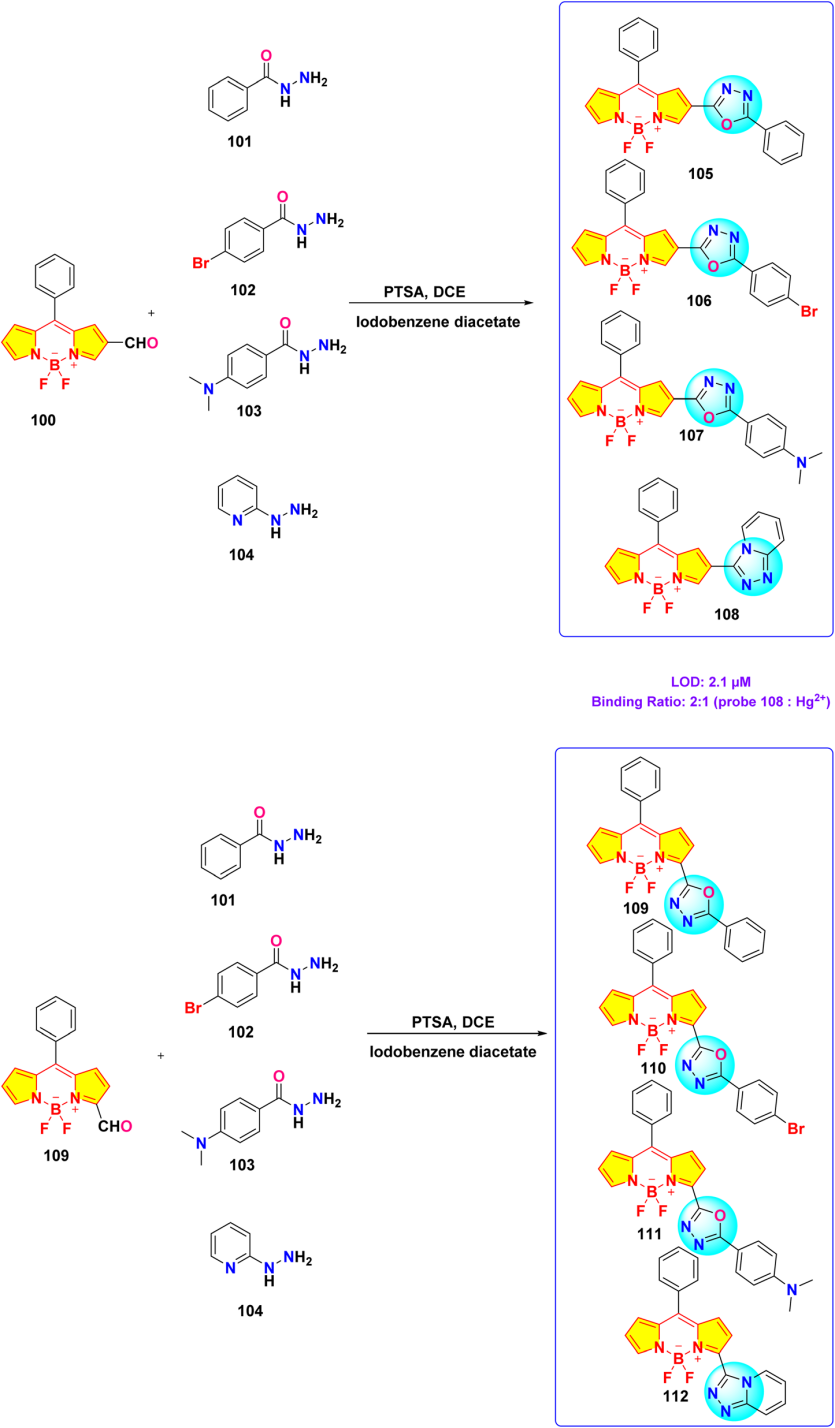
Synthetic route for probes 105–112.

In 2023, Kumar *et al.* synthesized a series of α/β isomeric oxadiazolyl-BODIPY derivatives (probe 105–112) for the selective detection of Hg^2+^ ions (Scheme 16).^105^ While no significant changes were observed in the absorption spectra of compounds 105–112 upon the addition of various metal ions, probe 108 exhibited a rapid and specific response towards Hg^2+^ ions in methanolic solution. Upon Hg^2+^ ion addition, the absorption spectra of all compounds displayed a shift towards longer wavelength in the absorption maxima accompanied by a decrease in the extinction coefficient. The probe 108 demonstrated a binding constant of 1.8 × 10^4^ M^−1^ and a LOD of 2.1 μM. Job's plot analysis revealed a 2 : 1 binding stoichiometry between the probe 108 and Hg^2+^ ions. Notably, compound 108 exhibited enhanced fluorescence intensity upon Hg^2+^ ion binding, attributed to the suppression of the photoinduced electron transfer (PET) process.

## 4. Conclusion and future outlook

**Fig. 25:**
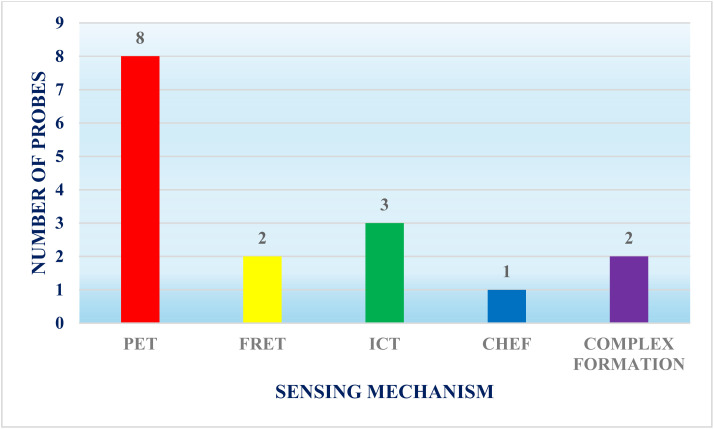
Different sensing mechanisms utilized by BODIPY-based chemosensors for the detection of mercury ions.

The advancement in the development of BODIPY-based fluorescent sensors for the detection of mercury ions (Hg^2+^) signifies noteworthy progress in tackling the global issue of mercury pollution. Over the past six years (2019–2024), innovative synthetic strategies and photophysical mechanisms have enhanced the sensitivity, selectivity, and practicality of these sensors (Table 1). This analysis constitutes the first exhaustive review to systematically delineate the design principles, synthetic pathways, and sensing mechanisms of BODIPY-based architectures tailored for precise Hg^2+^ identification. Key advancements include the strategic incorporation of BODIPY fluorophores with macrocyclic receptors (*e.g.*, pillararenes), dual-channel architectures (*e.g.*, rhodamine-BODIPY hybrids), and sulfur-enriched ligands (*e.g.*, thiosemicarbazides), achieving detection limits at ultratrace levels across aqueous and aqueous–organic matrices. Functional moieties such as thiosemicarbazides, crown ethers, and pillararenes enhance selectivity and binding affinity by exploiting Hg^2+^'s coordination preferences. These sensors leverage mechanisms such as photoinduced electron transfer (PET), Förster resonance energy transfer (FRET), and chelation-enhanced fluorescence (CHEF) to enable real-time, on-site monitoring with minimal interference from competing ions (Fig. 25). A key advantage of BODIPY-based probes is their structural tunability, which enables ratiometric signaling, colorimetric responses, and near-infrared (NIR) emission. For example, integrating soft donor atoms, such as sulfur or nitrogen, into macrocyclic or thiourea frameworks can exploit soft acid–soft base interactions, which are particularly beneficial for selectively binding mercury ions over other metal ions. Additionally, steric hindrance can be introduced around the binding site to minimize non-specific interactions. Meanwhile, ratiometric or dual-mode sensing approaches, which combine both colorimetric and fluorometric responses, provide built-in calibration that further discriminates against interfering species. Advances in computational modeling and machine learning also play a crucial role in predicting binding affinities and guiding the design of receptors that achieve a delicate balance between sensitivity and selectivity. Collectively, these approaches pave the way for developing high-performance sensors with enhanced selectivity in complex sample matrices.

**Table 1:** Summary of BODIPY-based molecules for the detection of mercury ions

Sl no.	Probe	Optical response	Solvent	LOD	Stoichiometric ratio	Colour change	Mechanism	Ref.
1	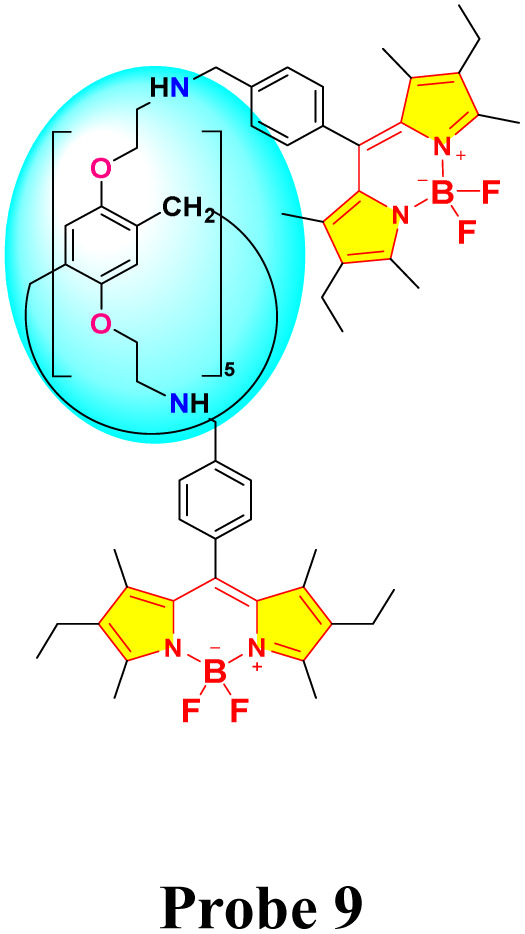	Turn-off	CH_3_CN : H_2_O (1 : 1)	0.2 μM	1 : 2	—	PET	78
2	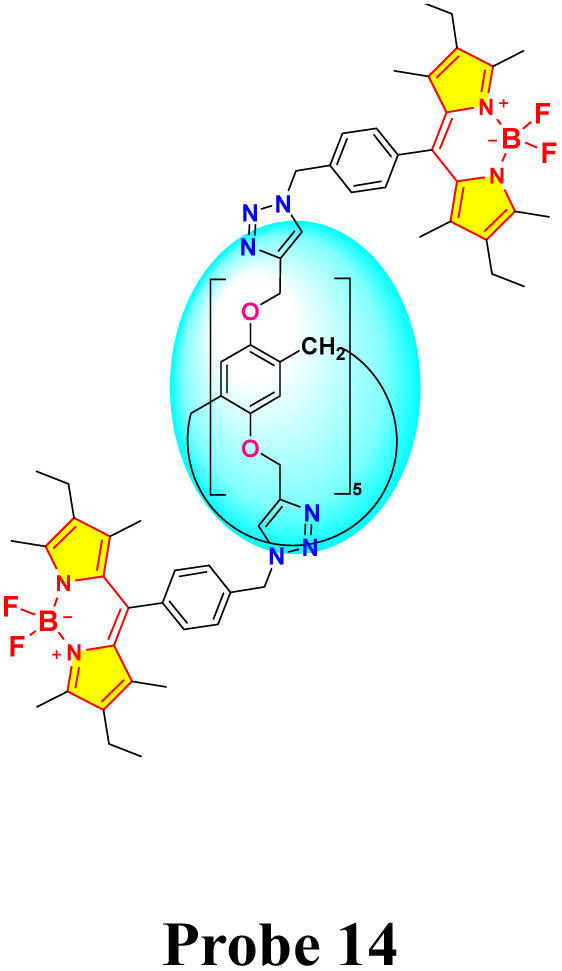	Turn-off	CH_3_CN : H_2_O (1 : 1)	1.09 μM	1 : 2	Green-deep green	PET	62
3	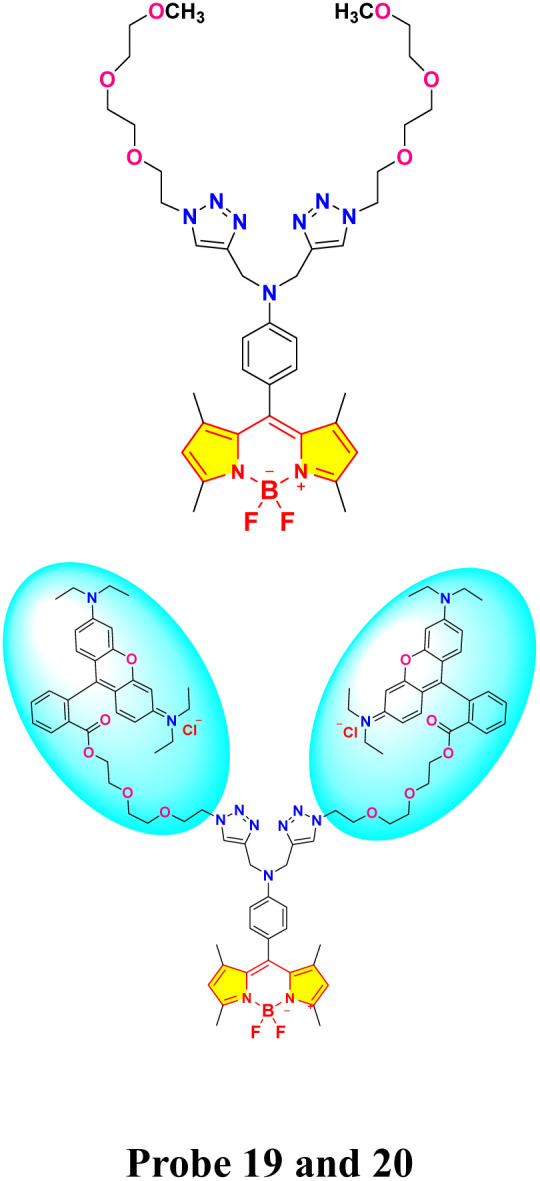	Turn-on	CH_3_CN : H_2_O (9 : 1)	0.01 μM	1 : 2	Colourless-green	FRET	81
				0.44 μM		Colourless-orange		
4	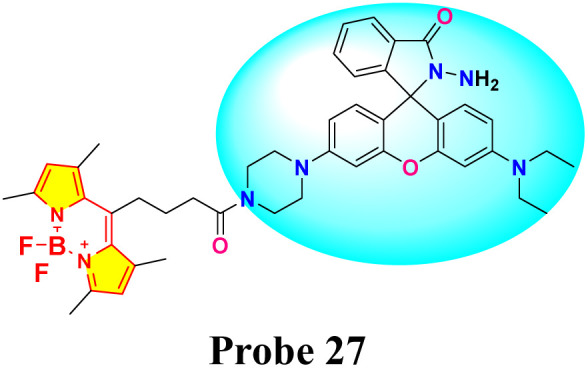	Turn-on	MeOH : H_2_O (7 : 3)	0.3 μM	—	Yellow-pink	FRET	82
5	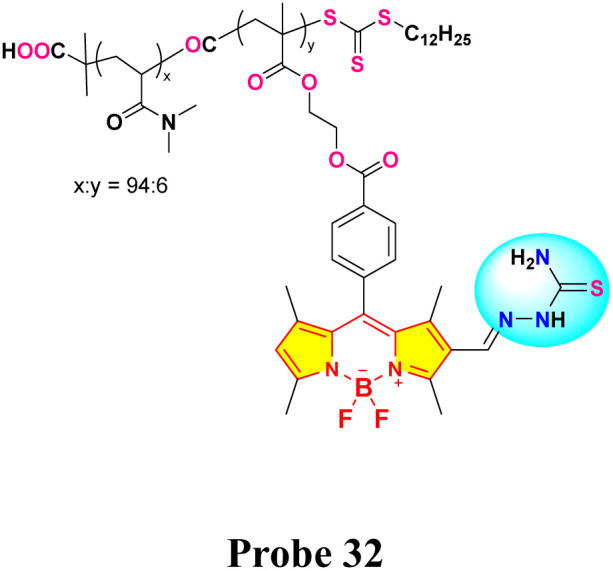	Turn-on	H_2_O	0.37 μM	2 : 1	Blue-yellow	Complex formation	84
6	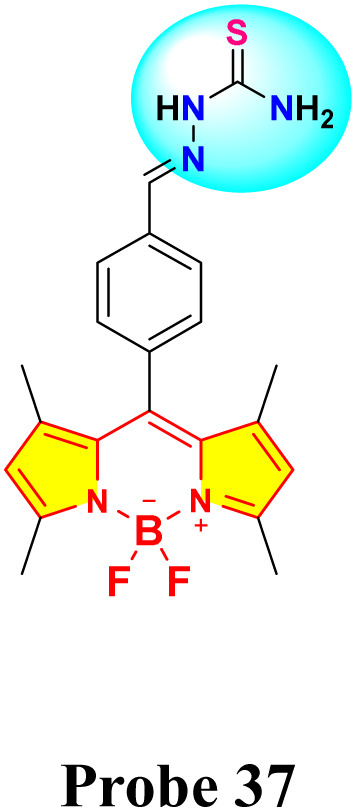	Turn-off	H_2_O : DMF (8 : 2)	0.49 μM	1 : 1	Green-colourless	Complex formation	44
7	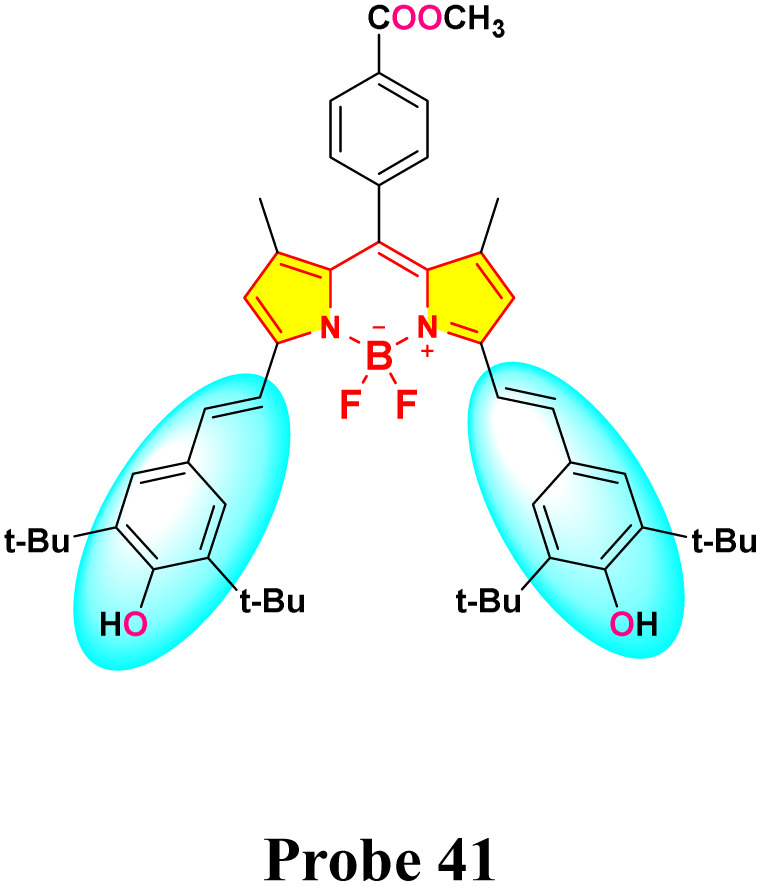	Turn-off	Aq. THF	0.7 μM	1 : 2	Green-light yellow	Inhibition of ICT	91
8	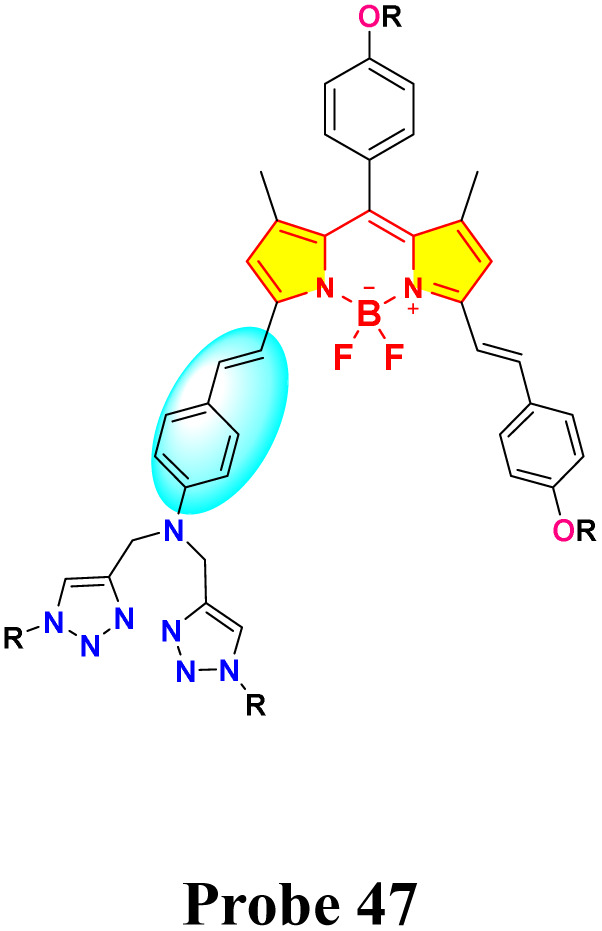	Turn-on	CH_3_CN : H_2_O (9 : 1)	0.09 μM	1 : 2	Green-blue green	Inhibition of PET	92
9	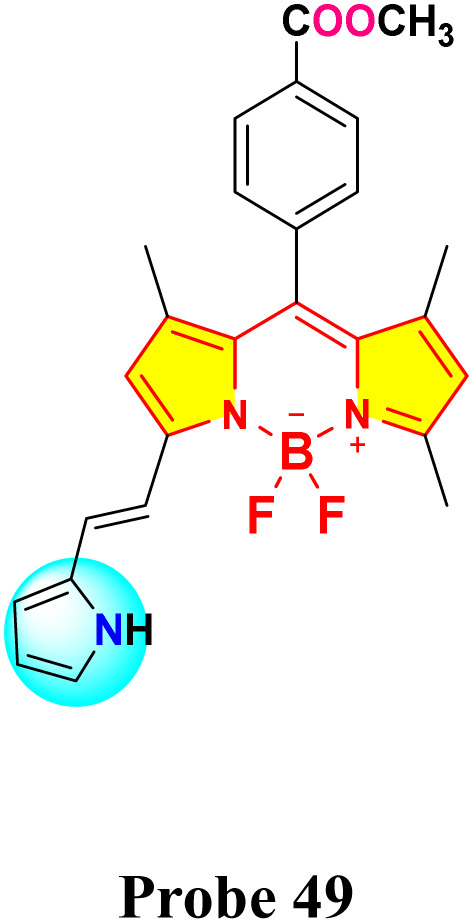	Turn-off	Aq. THF	0.05 μM	1 : 2	Pink-colourless	Inhibition of ICT	93
10	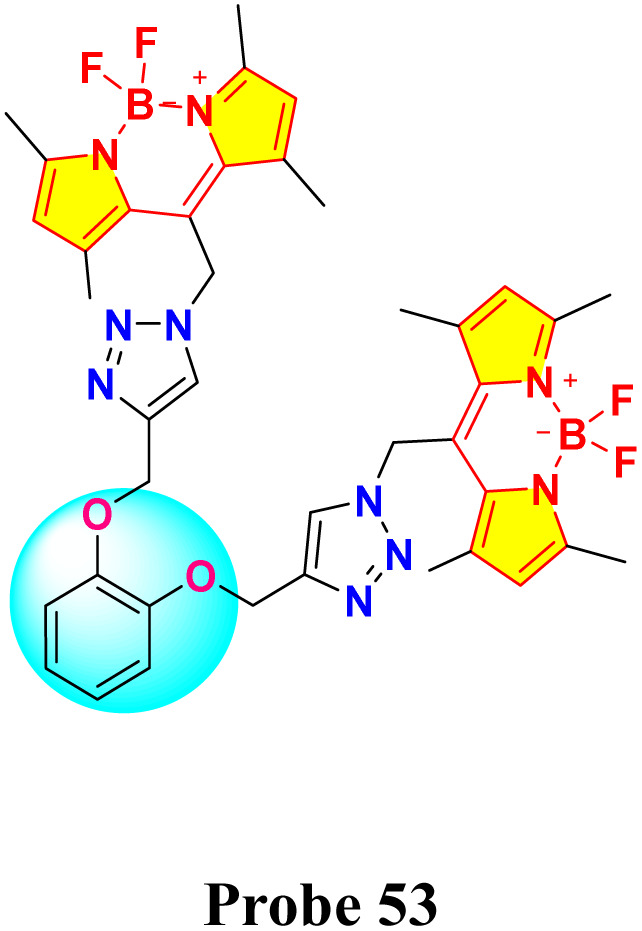	Turn-on	MeOH	1 μM	1 : 1	—	CHEF	99
11	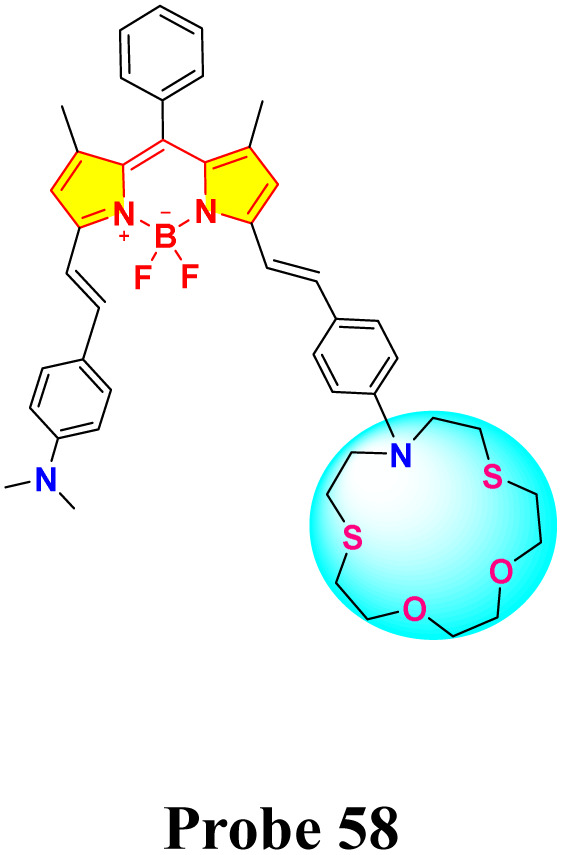	Turn-on	DMF : H_2_O (3 : 7)	26.6 μM	1 : 1	Light Brown-green	Inhibition of PET	100
12	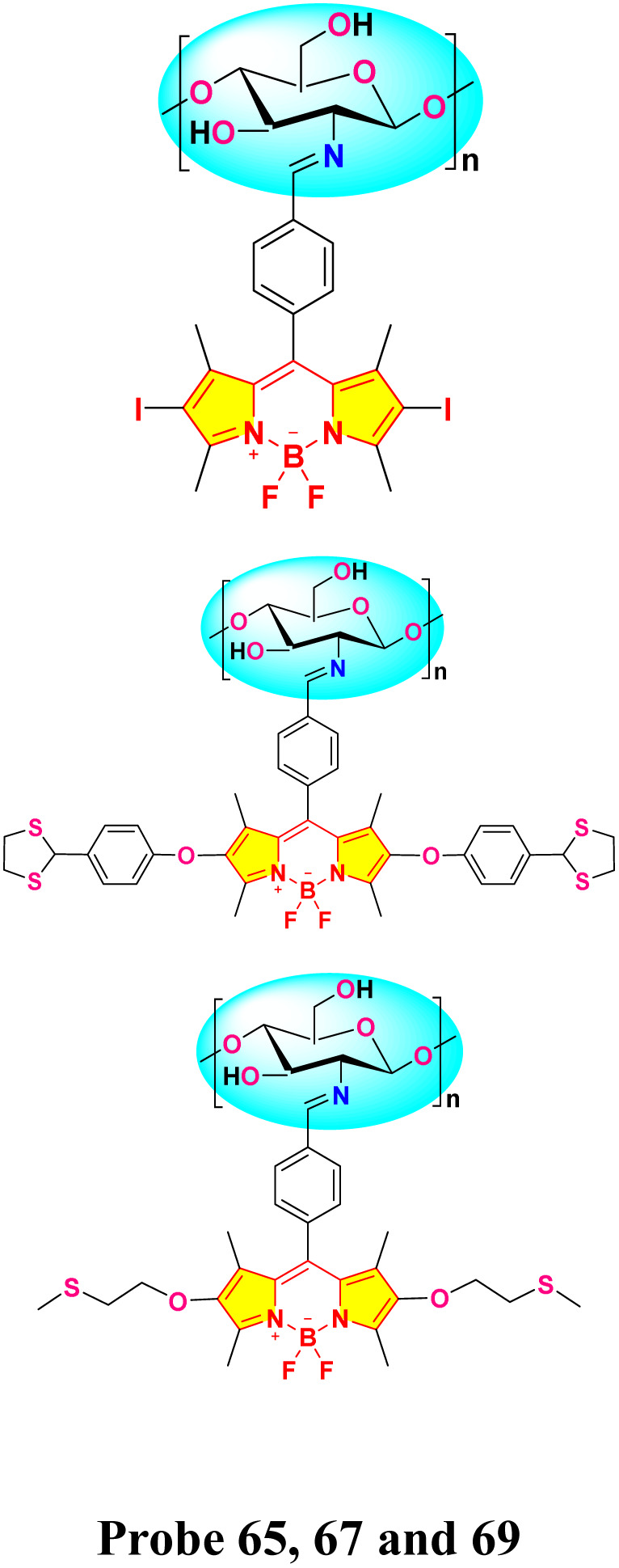	Turn-off	Aq. Acetic acid	1.51 μM (65-Hg^2+^)	—	—	PET	101
				1.53 μM (65-Hg^+^)				
				1.61 μM (67-Hg^2+^)				
				2.00 μM (67-Hg^+^)				
				3.24 μM (69-Hg^2+^)				
				2.79 μM (69-Hg^+^)				
13	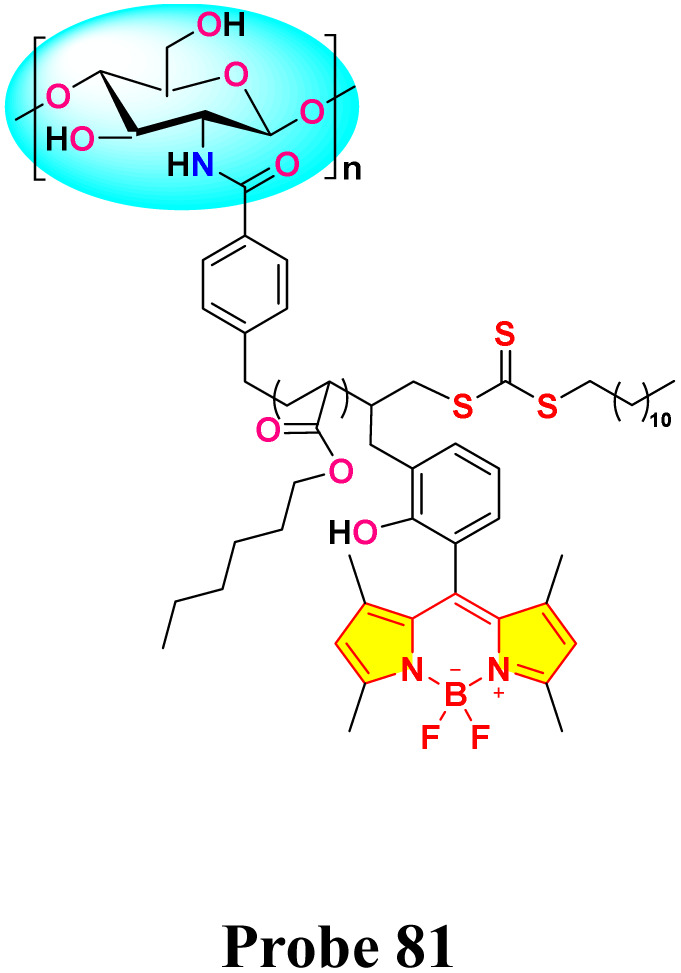	Turn-on	Organic solvent	0.61 μM (Hg^2+^)	—	—	PET	102
				0.47 μM (Hg^+^)				
14	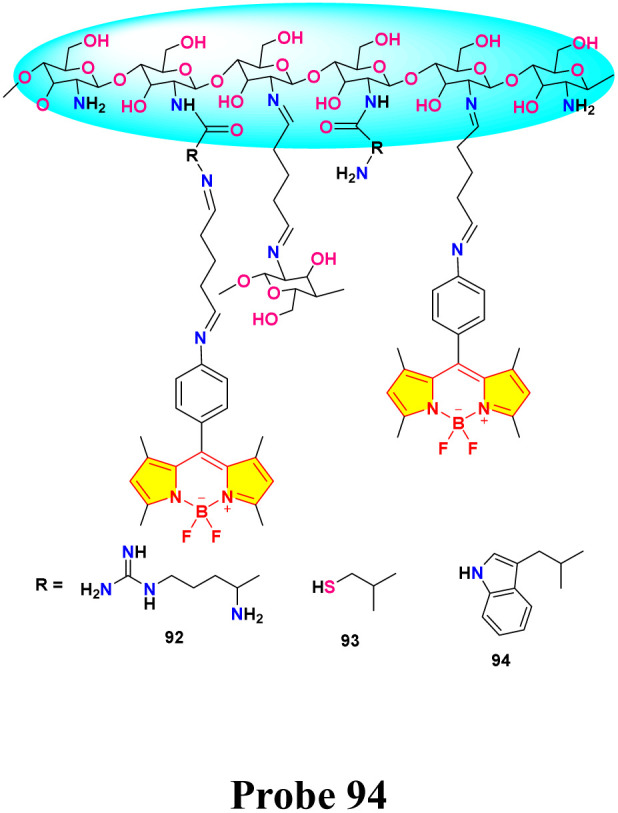	Turn-off	1% acetic acid	1.517 μM (Hg^2+^)	—	Orange-red	Inhibition of ICT	103
				1.357				
				μM (Hg^+^)				
				3.630 μM (Hg^2+^)				
15	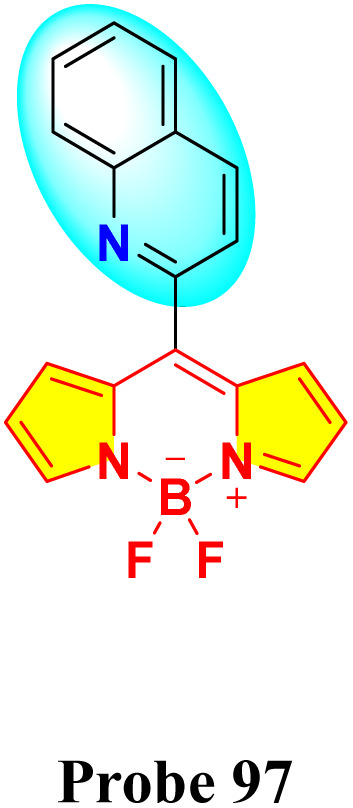	Turn-off	CH_3_CN : H_2_O (7 : 3)	0.03 μM	1 : 1	—	PET	104
16	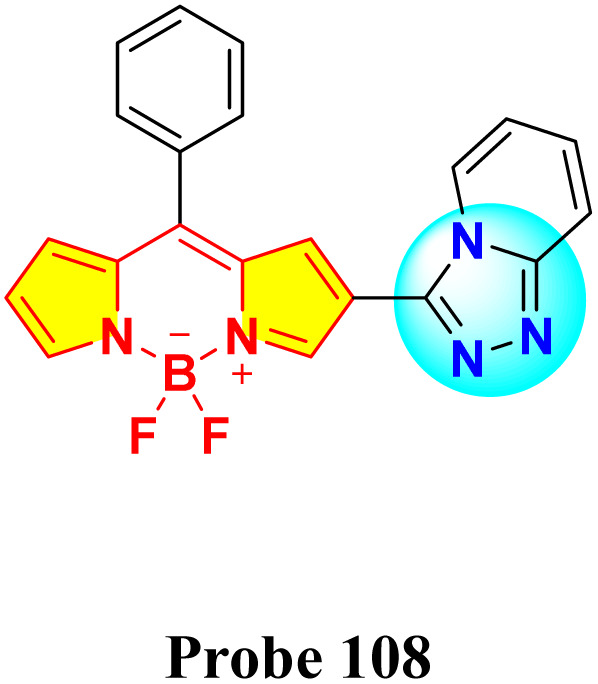	Turn-on	MeOH	2.1 μM	2 : 1	—	Inhibition of PET	105

Despite these breakthroughs, challenges persist in optimizing sensor stability across diverse pH ranges and mitigating matrix effects in complex samples. Furthermore, interference caused by coexisting ions (*e.g.*, Cu^2+^, Fe^3+^) and organic compounds present in complex samples compromises selectivity, leading to erroneous signals or fluorescence quenching. Future research should focus on enhancing biocompatibility for *in vivo* imaging, developing recyclable sensor materials, and integrating these systems into portable devices for field applications. Integration of BODIPY-based sensors with intelligent devices, such as portable photometers,^106–110^ portable Raman meters,^111–114^ and mobile phones,^19,115,116^ can provide quick and precise data collection in real-time, streamline detection processes, and offer high practicality. Additionally, incorporating BODIPY-based sensors into testing papers offers an extremely cost-effective method for detecting metal ions.^117–121^ Furthermore, BODIPY-based sensors integrated into hydrogels^122–126^ or thin films^127^ could unlock better sensing capabilities and expand their practical applications. By addressing these challenges and building on existing knowledge, BODIPY-based sensors hold significant potential for meeting the growing demand for efficient and reliable metal ion detection.

## Data availability

No primary research results, software or code have been included and no new data were generated or analysed as part of this review.

## Conflicts of interest

The authors report no conflicts of interest. The authors alone are responsible for the content and writing of this article.
